# Re-programming mouse liver-resident invariant natural killer T cells for suppressing hepatic and diabetogenic autoimmunity

**DOI:** 10.1038/s41467-022-30759-w

**Published:** 2022-06-07

**Authors:** Channakeshava Sokke Umeshappa, Patricia Solé, Jun Yamanouchi, Saswat Mohapatra, Bas G. J. Surewaard, Josep Garnica, Santiswarup Singha, Debajyoti Mondal, Elena Cortés-Vicente, Charlotte D’Mello, Andrew Mason, Paul Kubes, Pau Serra, Yang Yang, Pere Santamaria

**Affiliations:** 1 Julia McFarlane Diabetes Research Centre and Department of Microbiology, Immunology and Infectious Diseases, Snyder Institute for Chronic Diseases and Hotchkiss Brain Institute, Cumming School of Medicine, University of Calgary, Calgary, Alberta T2N 4N1 Canada; 2 Institut D’Investigacions Biomèdiques August Pi i Sunyer, Barcelona, 08036 Spain; 3 Department of Physiology and Pharmacology and Snyder Institute for Chronic Diseases, Cumming School of Medicine, University of Calgary, Calgary, Alberta T2N 4N1 Canada; 4 Division of Gastroenterology and Hepatology, University of Alberta, 7-142 Katz Group Rexall Centre, Edmonton, T6G 2E1 Canada; 5 Department of Biochemistry and Molecular Biology and Snyder Institute for Chronic Diseases, Cumming School of Medicine, University of Calgary, Calgary, Alberta T2N 4N1 Canada

**Keywords:** Innate lymphoid cells, Nanoparticles, Autoimmunity, B cells

## Abstract

Invariant NKT (iNKT) cells comprise a heterogeneous group of non-circulating, tissue-resident T lymphocytes that recognize glycolipids, including alpha-galactosylceramide (αGalCer), in the context of CD1d, but whether peripheral iNKT cell subsets are terminally differentiated remains unclear. Here we show that mouse and human liver-resident αGalCer/CD1d-binding iNKTs largely correspond to a novel Zbtb16^+^Tbx21^+^Gata3^+^Maf^low^Rorc^–^ subset that exhibits profound transcriptional, phenotypic and functional plasticity. Repetitive in vivo encounters of these liver iNKT (LiNKT) cells with intravenously delivered αGalCer/CD1d-coated nanoparticles (NP) trigger their differentiation into immunoregulatory, IL-10+IL-21-producing Zbtb16^high^Maf^high^Tbx21^+^Gata3^+^Rorc^–^ cells, termed LiNKTR1, expressing a T regulatory type 1 (TR1)-like transcriptional signature. This response is LiNKT-specific, since neither lung nor splenic tissue-resident iNKT cells from αGalCer/CD1d-NP-treated mice produce IL-10 or IL-21. Additionally, these LiNKTR1 cells suppress autoantigen presentation, and recognize CD1d expressed on conventional B cells to induce IL-10+IL-35-producing regulatory B (Breg) cells, leading to the suppression of liver and pancreas autoimmunity. Our results thus suggest that LiNKT cells are plastic for further functional diversification, with such plasticity potentially targetable for suppressing tissue-specific inflammatory phenomena.

## Abstract (web summary)

Invariant natural killer T (iNKT) cells are tissue-resident immune cells recognizing lipid antigens. Here the authors find that liver, but not lung nor spleen, iNKT cells alter their transcriptome upon systemic treatment of lipid nanoparticles for the induction of regulatory B cells and suppression of liver and pancreas autoimmunity in mouse models.

## Introduction

Type 1 or invariant NKT (iNKT) cells comprise a subset of T lymphocytes that express a semi-invariant TCR and recognize glycolipids presented by CD1d^1^. iNKT cells develop in the thymus and exist as five major subsets with distinct cytokine and transcription factor expression profiles. They are poised to rapidly produce significant amounts of Th1- (IFNγ), Th2 (IL-4 and IL-10), Th10 (IL-10), Th17 (IL-17), or T-Follicular Helper (IL-21)-type cytokines upon activation, and have been implicated not only in anti-microbial and anti-tumor immunity, but also in the pathogenesis and regulation of a variety of inflammatory disorders, such as autoimmune diseases^2–5^.

Upon thymic development and differentiation, iNKT cell subsets distribute to different tissues with varying frequencies, and do not migrate through the bloodstream. Although tissue-bound iNKT thymic emigrants further specialize at their site of residence, it is generally believed that tissue-resident iNKT cell subsets are terminally differentiated^4–11^. Although iNKT10 and iNKT-FH cells have not been documented in the thymus and thus may arise in the periphery from as yet undefined precursors, it remains possible that they expand from small pools of pre-differentiated, thymic-derived cells^1^. Thus, whether peripheral iNKT cells are terminally differentiated or re-programmable remains unclear. Addressing this question would help provide new insights into if and how these cells can be manipulated for therapeutic purposes^1^.

In sterile liver injury, liver-resident iNKT cells promote tissue repair^12^. On the other hand, αGalCer-induced activation of iNKT cells can induce liver damage^13,14^ and microbial activation of iNKT cells can trigger liver autoimmunity^15^. iNKT cells were also shown to play a critical role in the pathogenesis of Concanavalin A-induced hepatitis^16^, alcoholic liver disease^17^, non-alcoholic steatohepatitis (NASH), as well as ischemia-reperfusion^18^, viral-induced hepatitis (HBV/HBC) and drug-induced liver injuries^19^. Furthermore, the frequency of liver iNKT cells in human Primary Biliary Cholangitis (PBC) is elevated, and introduction of a genetic iNKT cell deficiency into a mouse model of PBC significantly decreased pathology^20^. Thus, the liver iNKT cell pool has tissue repair or pro-inflammatory properties in a context-dependent manner, suggesting that it is either plastic or composed of a heterogeneous mixture of thymus-derived subsets that differentially expand or wane in response to various physiological or pathological cues.

We have shown that NPs coated with mono-specific autoimmune disease-relevant peptide-major histocompatibility complex class II (pMHCII) molecules^21^ can re-program cognate autoantigen-experienced CD4 + T cells into FoxP3^–^CD25^–^ TR1 cells via direct and sustained ligation of their antigen receptors^21^. This is followed by systemic expansion, local recruitment and generation of organ-specific regulatory cell networks capable of reverting various experimental and spontaneous organ-specific autoimmune diseases in mice^22–25^. Here, we have engineered αGalCer/CD1d-NPs to investigate if the (potentially plastic) liver-resident αGalCer/CD1d-binding iNKT cells can be re-programmed into liver-autoimmune disease-suppressing cells. Since intravenously-delivered MHC-coated NPs are rapidly recruited and transiently retained in the large liver sinusoidal vascular bed^21^, and since liver-resident iNKT cells, unlike other tissue-resident iNKT cells, regularly patrol the liver vasculature^12^, we reasoned that these compounds would readily be able to ligate cognate TCRs on these T cells.

We find that the liver-resident iNKT cell subset, both in humans and mice, largely corresponds to one subset that has a clearly distinct transcription factor-expression profile as compared to all other thymic and peripheral iNKT cell subsets described to date. Remarkably, upon αGalCer/CD1d-NP treatment, this prevalent subset locally differentiates into yet another subset that acquires TR1-like transcriptional, phenotypic and functional properties, including an ability to orchestrate the formation of a local immunoregulatory cell network that blunts various forms of liver autoimmunity and can reverse spontaneous hyperglycemia in nonobese diabetic mice. Thus, the LiNKT subset is plastic and such plasticity can be harnessed for tissue-specific therapeutic intervention in autoimmunity.

## Results

### αGalCer/CD1d-NPs blunt liver autoimmunity

**Fig. 1:**
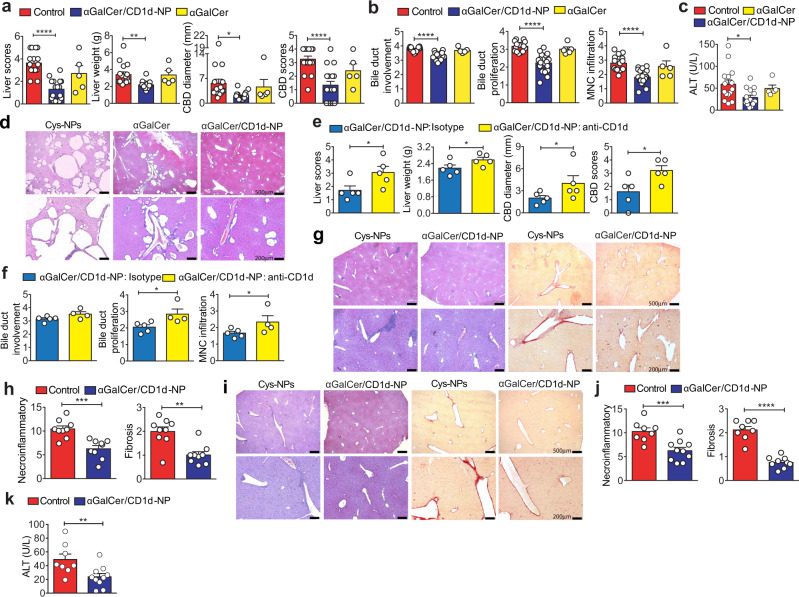
αGalCer/CD1d-NP-treatment blunts liver autoimmunity. **a** Macroscopic PBC scores in mice treated with Cys-NPs or left untreated (controls, pooled as no differences were seen between the two: *n* = 16; treated = 12; untreated = 4; 4 experiments), αGalCer/CD1d-NPs (*n* = 18; 4 experiments) or αGalCer (*n* = 5). **b** Microscopic PBC scores corresponding to control NOD.*c3c4* mice (*n* = 16; treated with Cys-NPs = 12; untreated = 4; 4 experiments), and NOD.*c3c4* mice treated with αGalCer/CD1d-NPs (*n* = 21; 4 experiments) or αGalCer (*n* = 5). **c** Serum ALT levels in control NOD.*c3c4* mice (*n* = 16; treated with Cys-NPs = 12; untreated = 4; 4 experiments), and NOD.*c3c4* mice treated with αGalCer/CD1d-NPs (*n* = 20; 4 experiments). **d** Representative images of H&E liver sections corresponding to NOD.*c3c4* mice from panel **b**. Scale bars, 500 μm (top) and 200 μm (bottom). **e**, **f** Average gross (**e**) and microscopic (**f**) PBC scores in NOD.*c3c4* mice treated with αGalCer/CD1d-NPs and rat IgG1 isotype or anti-CD1d mAb (*n* = 5 in each group). **g**, **h** Representative images of H&E- and Picrosirius Red-stained liver sections (**g**) and average microscopic PBC scores (**h**) for (NODxB6.*Ifng-ARE-Del*^*–/–*^) F1 mice treated with Cys-NPs (*n* = 9; 3 experiments) or αGalCer/CD1d-NPs (*n* = 9; 3 experiments). Scale bars, 500 μm (top) and 200 μm (bottom). **i**, **j** Representative images of H&E- and Picrosirius Red-stained liver sections (**i**) and average microscopic AIH scores (**j**) from NOD mice with Ad-FTCD-induced AIH, treated with Cys-NPs (*n* = 8; 2 experiments) or αGalCer/CD1d-NPs (*n* = 10; 3 experiments). Scale bars, 500 μm (top) and 200 μm (bottom). **k**, Serum ALT levels (*n* = 8 and 10, treated with Cys-NPs and αGalCer/CD1d-NPs, respectively. Data are represented as means ± SEM and were compared using one-way ANOVA with Tukey’s multiple comparison test (**a**–**c**) or one tail Mann–Whitney *U* test (**e**, **f**, **h**, **j**, **k**). *P < 0.05; **P < 0.01; ***P < 0.001; ****P < 0.0001.

Treatment of NOD.*c3c4* mice with αGalCer/CD1d-coated NPs, but not αGalCer alone or Cys-coated (control) NPs suppressed the progression of spontaneous PBC in this strain (Fig. 1a–d). In vivo CD1d blockade inhibited these effects (Fig. 1e, f). Similar results were obtained in B6 mice carrying a deletion of the IFNγ 3′-untranslated region adenylate uridylate-rich element (ARE) (ARE-Del^+/–^), which develop a form of cholangitis that, like human PBC, is accompanied with liver fibrosis^26^ (Fig. 1g, h). αGalCer/CD1d-NPs also had therapeutic activity in NOD mice with experimental autoimmune hepatitis (AIH)^27^ (Fig. 1i–k).

### αGalCer/CD1d-NPs promote liver iNKT cell activation and expansion

**Fig. 2:**
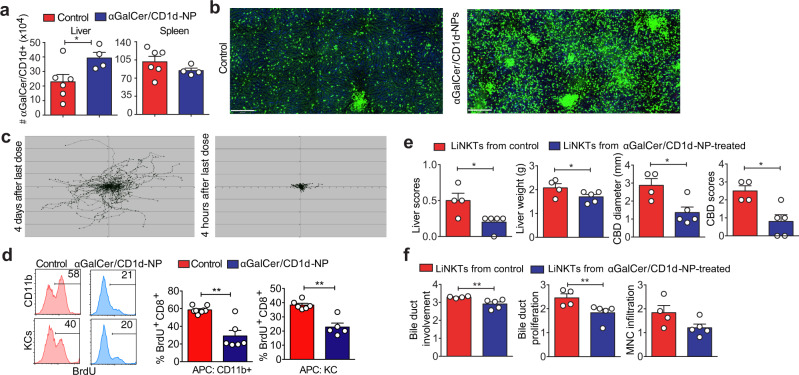
αGalCer/CD1d-NPs activate, expand and elicit immunoregulatory properties on liver iNKT cells. **a** Absolute numbers of αGalCer/CD1d tetramer+ iNKT cells in the liver and spleen of control NOD.*c3c4* mice (*n* = 6, untreated) vs. NOD.*c3c4* mice treated with αGalCer/CD1d-NPs (*n* = 4). Cells were enumerated 2–3 days after the last αGalCer/CD1d-NP dose. **b** Images depicting increased accumulation of Cxcr6^GFP/+^ cells in the liver in response to αGalCer/CD1d-NP vs. Cys-NP treatment of B6.*Ifng-Delta-ARE*^+/-^.*Cxcr6*^*GFP/+*^ mice. **c** Migration paths of Cxcr6^GFP/+^ cells in the livers of these mice within 4 days of the 10th dose and 4 h of an αGalCer/CD1d-NP dose. **d** Representative FACS histograms and average percentages of BrdU incorporation by splenic 8.3-CD8 + T cells in response to NRP-V7 peptide-pulsed PCLN CD11b + or liver KCs purified from Cys-NP- or αGalCer/CD1d-NP-treated mice. Data correspond to *n* = 8 and 6 (2 experiments) in the left panel and *n* = 6 and 5 (2 experiments) in the right panel. **e**, **f** Evaluation of gross (**e**) and microscopic (**f**) PBC scores in NOD.*c3c4.scid* hosts transfused with iNKT cells isolated from the livers of Cys-NP- or αGalCer/CD1d-NP-treated mice followed, 1 day later, by transfusion of whole splenocytes and PCLN cells from diseased NOD.*c3c4* donors. Data correspond to *n* = 4/group (red) and 5/group (blue) from left to right. Data are represented as means ± SEM and were compared using one tail Mann–Whitney *U* test (**a**, **d**–**f**). *P < 0.05; **P < 0.01; ***P < 0.001; ****P < 0.0001.

αGalCer/CD1d-NP treatment increased the absolute numbers of αGalCer/CD1d tetramer+ iNKT cells in the liver but not the spleen of NOD.*c3c4* mice, suggesting a liver-specific effect (Fig. 2a). We used spinning-disk confocal intravital microscopy to visualize the effects of αGalCer/CD1d-NPs on the behavior of liver iNKT (LiNKT) cells in Cxcr6^GFP/+^ B6.*Ifng-Delta-ARE*^+/-^ mice, in which a significant fraction of liver eGFP+ cells are iNKTs^12^. Treatment with αGalCer/CD1d-NPs for 5 weeks triggered an increase in the number of intra-hepatic eGFP+ cells vs. controls (Fig. 2b). When imaged 4 days after the last dose, these eGFP+ cells (including both LiNKT and non-LiNKT cells) were randomly distributed throughout the liver vasculature and were highly motile (Fig. 2c). However, when imaged within 6 h, they were static, consistent with αGalCer/CD1d-induced activation of eGFP+ LiNKT cells^28^ and suppression of the motility of non-LiNKT eGFP+ cells (Fig. 2c).

### αGalCer/CD1d-NPs elicit immunoregulatory properties on LiNKT cells

The liver F4/80 + (largely Kupffer cells, KCs) and CD11b + APCs (largely macrophages and myeloid-derived dendritic cells) isolated form the livers and liver-draining lymph nodes (LNs) of αGalCer/CD1d-NP-treated mice could not adequately trigger the proliferation of diabetogenic TCR-transgenic CD8 + (8.3-CD8 + ) T cells^29,30^ ex vivo vs. APCs isolated from control mice (Fig. 2d). Coupled with the ability of LiNKT cells isolated from αGalCer/CD1d-NP-treated vs control mice to suppress the ability of peptide-pulsed KCs to activate cognate CD8 + T cells in vitro (see Fig. 6c below), these data suggested that the LiNKTs of αGalCer/CD1d-NP-treated mice had acquired immunoregulatory properties. In agreement with this, LiNKT cells isolated from αGalCer/CD1d-NP-treated (but not control) NOD.*c3c4* mice suppressed disease development in NOD.*c3c4.scid* hosts reconstituted with splenocytes from diseased NOD.*c3c4* donors (Fig. 2e, f).

### LiNKT cells have a unique transcriptional profile

To gain an unbiased insight into the nature of these LiNKT cells, we profiled the transcriptomes of sorted αGalCer/CD1d-binding LiNKT cells from untreated NOD.*c3c4*, NOD and C57BL/6 mice via RNAseq (Supplementary Fig. 1a provides representative flow sorting profiles). We focused our initial analyses on a relatively small number of genes (*n* = 41) whose expression varies among various iNKT cell subsets, including iNKT1, iNKT2, iNKT17, iNKT10 ^1,4,5,9,10,31,32^, lymph node FoxP3+ iNKT^33,34^, follicular-helper iNKT^35^, adipose-tissue-resident iNKT^9,36^, or Breg-induced iNKT cells^37^ (Table 1).

**Table 1:** Gene expression differences for iNKT cell markers and on known iNKT cell subsets vs. LiNKTs and LiNKTR1 cells.

Genes (protein name)	iNKT1^a^	iNKT2^a^	iNKT17^a^	iNKT10^a^	FoxP3+ iNKT^a^	ATR iNKT^a^	Breg- induced iNKT^a^	iNKT- FH^a^	C57BL/6 Liver iNKT^b^	NOD Liver iNKT^b^	NOD.*c3c4* Liver iNKT^b^	NOD.*c3c4* Liver iNKT treated^b^
*Zbtb16* (Plzf)	+/−	+	+	–	+	–	+	?	++++	++++	++++	++++
*Tbx21* (Tbet)	+	–	–	?	?	?	?	?	++++	++++	++++	++++
*Gata3* (Gata-3)	+/−	+	+	?	?	?	?	?	++++	++++	++++	++++
*Rorc* (Rorgt)	–	–	+	?	?	?	?	?	+/−	–	+	+
*Foxp3* (Foxp3)	–	–	–	–	+	–	–	?	–	–	–	–
*Maf* (Maf)	–	–	+	?	?	?	?	?	++++	++++	+++	+++++
*Nfil3* (E4bp4)	–	–	–	+	?	+	–	?	++	+++	++++	++++
*Bcl6* (Bcl6)	?	?	?	–	?	?	?	+	++	++	+	+
*IRF4* (Irf4)	–	+	+	?	?	?	?	?	+++	+++	+++	++++
*Lef1* (Lef-1)	+	+	+	+	?	?	?	?	++	++	++	++
*Cd24a* (CD24)	+/−	+/−	+/−	+	?	?	?	?	+/−	+/−	+/−	+/−
*Cd44* (CD44)	+	+	+	?	?	?	?	?	+++++	+++++	+++++	+++++
*Klrb1b/c* (NK1.1)	+	–	+/−	+/−	?	+/−	?	?	++++	++	++	++
*Ifng* (IFNg)	+	–	–	?	?	?	?	?	++++	+++++	+++++	+++++
*Il2* (IL-2)	–	–	–	?	?	+	?	?	–	–	–	–
*Il4* (IL-4)	+	+	–	?	?	+	?	?	++	+	+	+++
*Il17a* (IL-17)	–	–	+	?	?	?	?	?	+/−	–	+/−	+/−
*Il10* (IL-10)	–	–	–	+	?	+	?	?	–	–	–	+++++
*Il21* (IL-21)	–	–	–	?	?	?	?	+	–	–	–	++++
*Il2rb* (CD122)	+	–	–	?	?	?	?	?	+++++	+++++	+++++	+++++
*Mirlet7* (Let-7)	+	?	?	?	?	?	?	?	–	–	–	–
*Zfp683* (Hobit)	+	?	?	?	?	?	?	?	++++	+++	++	++
*Il17rb* (IL-17 Rb)	–	+	+/−	?	?	?	?	?	–	–	+/−	–
*Tnfsf11* (Rankl)	–	+	+	?	?	?	?	?	+	++	+	+
*Tnfrsf4* (CD134)	?	?	?	+	?	?	?	?	–	–	–	+++
*Il7r* (IL-7R)	?	?	?	+	?	?	?	?	+++	+++	+++	++++
*Tgfbr2* (Tgfbr2)	?	?	+	?	?	?	?	?	++++	++++	++++	++++
*Lag3* (Lag-3)	?	?	?	?	?	?	?	?	+	–	+/−	+++++
*Ctla4* (Ctla-4)	?	?	?	+	?	?	?	?	++	+	++	+++++
*Slamf6* (Slaf6)	–	+	–	+	?	?	?	?	+	++	+	+++
*Itga4* (CD49d)	?	?	–	+	?	?	?	?	+++	+++	+	++++
*Itgae* (CD103)	?	?	+	–	?	?	?	?	+/−	+	++	+
*Pdcd1* (Pd1)	?	?	?	+	?	?	?	+	–	+	+	+++
*Tigit* (Tigit)	?	?	?	?	?	?	?	?	+	+	+	++++
*Ccr6* (Ccr6)	–	–	+	?	?	?	?	?	–	+/−	+	–
*Ccr9* (Ccr9)	–	+	–	+	?	?	?	?	+++	+++	+++	+/−
*Cxcr5* (Cxcr5)	–	+	–	?	?	?	?	+	–	+/−	+/−	+/−
*ICOS* (Icos)	+/−	+	+	+	?	?	?	+	++++	++++	++++	++++
*Nrp1* (Nrp1)	?	?	?	+	?	?	?	?	–	+/−	+/−	+
*Izumo1r* (Fr4)	?	?	?	+	?	?	?	?	++	++	+	++++
*Cd27* (CD27)	+	+	–	?	?	?	?	?	+++	+++	+++	++++

^a^From references (–, not expressed; +/–, low; +, expressed) (1, 4, 5, 9, 10, 29, 30, 31, 32, 33, 34, 35, 38, 39).

^b^From our RNAseq data as a function of normalized counts (–, <5 normalized counts; +/–, 5–10; +, 10–25; ++, 25–50; +++, 50–100; ++++, 100–500; +++++, >500).

Although the LiNKT cells of untreated NOD.*c3c4* mice could not be classified into any of these subsets, based on these markers (Table 1), they shared the expression of significantly more genes with iNKT1-like cells (126/246 genes –51.2%–) than with iNKT2- or iNKT17-like cells (25/293 –8.5%– and 40/237 genes –16.9%–, respectively)^32^ (Supplementary Data 1).

Comparison of the transcriptome of these cells to those of LiNKT cells isolated from the healthy livers of NOD and B6 mice indicated that these gene expression differences were more related to the organ source of the iNKT cells than to the disease status of, or genetic differences between NOD.*c3c4* vs. other strains (Supplementary Figs. 1b–d and Supplementary Data 2–3): the expression levels of the iNKT cell subset-defining genes listed in Table 1^1,4,5,9,10,31–37^ were similar among the three strains (Supplementary Fig. 1e). Therefore, the LiNKT cells from NOD.c*3c4*, NOD, and B6 mice express a unique transcriptome.

### Re-programming of LiNKT cells by αGalCer/CD1d-NPs

**Fig. 3:**
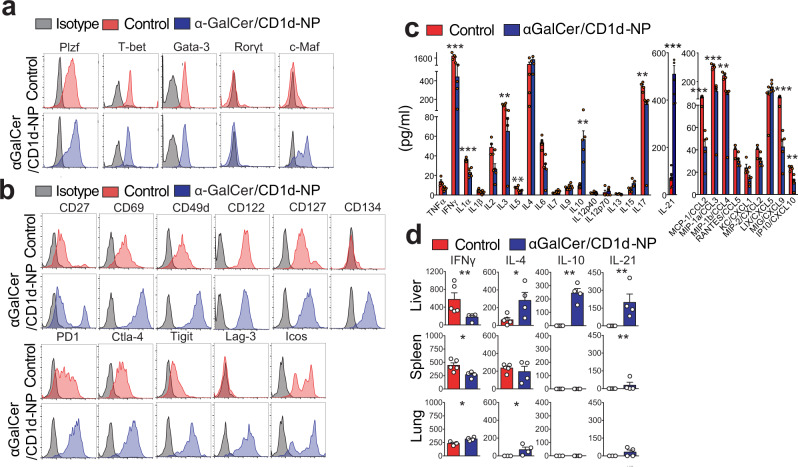
Phenotypic and cytokine profiles for control and αGalCer/CD1d-NP-challenged LiNKT cells. **a** Representative flow cytometric histogram profiles for TF staining in LiNKTs of control (*n* = 5, untreated) and αGalCer/CD1d-NP-treated NOD.*c3c4* mice (*n* = 4). **b** Representative flow cytometric histogram profiles for iNKT cell surface markers of control (*n* = 5, untreated) and αGalCer/CD1d-NP-treated NOD.*c3c4* mice (*n* = 4). **c** Cytokine and chemokine profiles of anti-CD3/anti-CD28 mAbs-stimulated LiNKT cells of αGalCer/CD1d-NP-treated (*n* = 5) vs. control NOD.*c3c4* mice (*n* = 4, Cys-NP-treated). **d** Secretion of IFNγ, IL-4, IL-10, and IL-21 by anti-CD3/anti-CD28 mAb-stimulated, αGalCer/CD1d tetramer+ cells isolated from the liver, spleen, or lungs of control (*n* = 5, untrated) and αGalCer/CD1d-NP-treated NOD.*c3c4* mice (*n* = 4). Data in **a**–**d** were generated using cells isolated 2–3 days after the last αGalCer/CD1d-NP dose. Data are represented as means ± SEM and were compared using one tail Mann–Whitney *U* test (**d**) or multiple *t*-test comparisons corrected using the Holm–Sidak method (**c**). *P < 0.05; **P < 0.01; ***P < 0.001; ****P < 0.0001.

We next compared the transcriptomes of the LiNKT cells of αGalCer/CD1d-NP-treated vs. control NOD.*c3c4* mice (Supplementary Fig. 1f–h and Supplementary Data 4–5). αGalCer/CD1d-NP-treatment triggered the upregulation of 162 genes in LiNKT cells, including genes encoding the transcriptional regulators Plzf and c-Maf, the immunoregulatory cytokines IL-10, IL-4, and IL-21 and the co-inhibitory receptors Lag-3, Ctla-4, PD-1, and Tigit, among others (Table 1 and Supplementary Fig. 2a). In addition, these LiNKT cells significantly downregulated 76 genes such as those encoding the chemokine receptors Ccr6, Ccr9 and the cytokine receptor IL-17Rβ (Table 1, Supplementary Fig. 2a, and Supplementary Data 4–5). Flow cytometric analyses for some of these markers were largely consistent with the transcriptional profiles (Fig. 3a, b, Table 1, and Supplementary Fig. 2b, c).

The LiNKT cells isolated from control NOD.*c3c4* mice secreted high levels of IFNγ and low levels of IL-4 and IL-17, but no or very low levels IL-10 or IL-21, upon stimulation with anti-CD3/anti-CD28 mAb-coated beads ex vivo (Fig. 3c). In contrast, the LiNKT cells from αGalCer/CD1d-NP-treated mice produced lower levels of IFNγ and IL-17 and secreted both IL-10 and IL-21 in response to the same stimuli (Fig. 3c). To ascertain whether the re-programming effects of αGalCer/CD1d-NP therapy on LiNKT cells were liver-specific or systemic, we compared the IFNγ, IL-4, IL10, and IL-21 cytokine secretion profiles of liver vs. splenic and lung-derived iNKT cells from the same mice via ELISA. In agreement with the Luminex data, the LiNKT cells from treated mice downregulated IFNγ, upregulated IL-4, and acquired the ability to secrete IL-10 and IL-21. In contrast, the cytokine profiles of the splenic and lung iNKT cells did not undergo significant changes in response to treatment, and neither of these iNKT cell types produced IL-10 or IL-21 (Fig. 3d).

Collectively, these data suggested that αGalCer/CD1d-NPs re-program LiNKT cells, but not their splenic or lung counterparts, into a novel immunoregulatory iNKT cell subset.

### αGalCer/CD1d-NPs induce a TR1-like transcriptional signature on LiNKT cells

αGalCer/CD1d-NP treatment increased the percentage of liver CD4 + iNKTs at the expense of CD4–CD8– iNKTs (Supplementary Fig. 2d). Interestingly, a number of the immunoregulatory molecules expressed by the LiNKT cells from αGalCer/CD1d-NP-treated mice were also found to be upregulated in the TR1-like CD4 + T cells that arise in NOD mice in response to pMHCII-NP therapy^22^. Specifically, of the 162 genes that were upregulated in LiNKT cells of αGalCer/CD1d-NP-treated vs. control mice, 110 (68%) were also upregulated in the cognate TR1-like CD4 + T cells of pMHCII-NP-treated mice as compared to conventional CD4 + T cells (Tconv; Supplementary Data 6). Furthermore, 95 of the 238 genes differentially expressed (162 upregulated and 76 downregulated) by the LiNKT cells of αGalCer/CD1d-NP-treated vs. control mice were co-expressed by the pMHCII-NP-induced TR1 cells (FDR > 0.01), including *Il10*, *Il21*, *Lag3*, *Pdcd1*, *Ctla4*, *Tigit*, *Tnfrsf8*, *Maf*, *Nfia*, and *Vdr*, among others. Supplementary Fig. 3 shows the normalized gene counts for the 66 genes that had >4-fold differences in gene expression in both LiNKT cells from treated vs. untreated mice and pMHCII-NP-induced TR1 vs. Tconv cells, showing highly similar levels of expression. Consequently, we call these αGalCer/CD1d-NP-induced LiNKT cells LiNKTR1 cells.

### De novo generation of LiNKTR1 cells from LiNKT cells

**Fig. 4:**
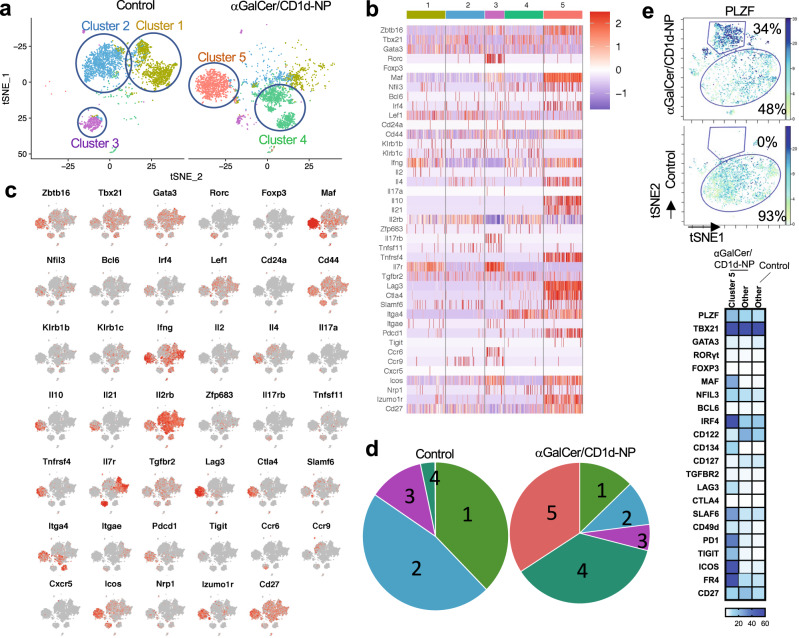
Single-cell RNAseq and mass cytometry of LiNKT cells in untreated vs αGalCer/CD1d-NP-treated mice. **a** tSNE plots from αGalCer/CD1d tetramer+ cells from the livers of two untreated (left) and two treated NOD.*c3c4* mice (right). Data for each individual mouse in each group were similar. Note how αGalCer/CD1d-NP therapy triggers a reduction in the size of clusters #1, #2, and #3, an increase in the size of cluster #4 and the de novo appearance of LiNKTR1 cells (Cluster #5). **b** Heatmaps comparing expression levels for the genes from Table 1 in the 5 liver iNKT cell clusters identified in untreated and/or αGalCer/CD1d-NP-treated mice. **c** Feature maps showing the location of gene expression for the genes from Table 1 and the combined cell clusters of untreated and treated mice (from **a**). **d** Pie charts depicting changes in the prevalence of the 5 LiNKT cell clusters in control vs. treated mice. **e** Cluster analyses of αGalCer/CD1d tetramer+ cells from the livers of three untreated and three treated NOD mice, upon staining with 22 metal-labeled antibodies and analysis via mass cytometry. Top panel shows a representative tSNE plot (for PLZF), indicating the position of the cluster 5 identified via scRNAseq (arising in response to αGalCer/CD1d-NP-treatment) and the iNKT cell pool comprising clusters 1–4 found in both treated and control mice (subpools cannot be resolved via CyTOF). Bottom panel, expression heatmaps for each marker in the LiNKTR1 scRNAseq cluster 5 vs. other LiNKT cells in treated mice (left, middle) and the LiNKT cells found in control mice (right). Data in **a**–**e** were generated using cells isolated 2–3 days after the last αGalCer/CD1d-NP dose.

To further define the identity of the LiNKTR1 subset that arises in response to αGalCer/Cd1d-NP treatment, we analyzed the scRNAseq profiles of LiNKT cells isolated from untreated and treated NOD.*c3c4* mice using the 10x genomics platform. The tSNE plots in Fig. 4a show the clustering of cells obtained from untreated and treated mice (2/condition; ~4000 cells/sample). In untreated mice, there were two major (#1 and #2) and a minor cluster of cells (#4) that shared the unique transcriptional signature of LiNKT cells identified via bulk RNAseq and thus probably represent alternative activation states of the same LiNKT cell subset (Fig. 4a, b and Table 2). There was also an additional, albeit small, cluster of cells (#3) with a clear iNKT17-like profile (Zbtb16^+^Tbx21^–^Gata3^+^Maf^+^Rorc^+^) (Fig. 4a, b and Table 2). Remarkably, αGalCer/CD1d-NP treatment elicited a major reduction in the size of clusters #1-3, a major increase in the size of cluster #4 and, importantly, the de novo appearance of a transcriptionally homogeneous iNKT cell cluster (#5; ~34% of cells) that co-expressed the LiNKT markers of Clusters #1 and #2, but co-upregulated the LiNKTR1 genes found to be upregulated in the bulk RNAseq dataset (Fig. 4a–d and Table 2). These results were further substantiated using mass cytometry, using a panel of 22 LiNKT and LiNKTR1-relevant antibodies. As shown in Fig. 4e and Supplementary Fig. 4, LiNKT cells from αGalCer/CD1d-NP-treated NOD mice contained both the LiNKTR1 cluster #5 identified via scRNAseq (~34% of cells), which was absent in control NOD mice, and a larger cluster of cells corresponding to the scRNAseq subclusters #1-2 and #4. As expected, based on the lack of therapeutic activity of αGalCer lipid against liver autoimmunity (Fig. 1a–d), αGalCer lipid was significantly less (~4-fold) efficient at inducing LiNKTR1 cell formation than αGalCer/CD1d-NPs, as measured via mass cytometry (Supplementary Fig. 5a). In addition, the few LiNKTR1 cells arising in αGalCer-treated mice expressed significantly different levels of some of the LiNKTR1 markers measured in this assay, such as the LiNKTR1-associated transcription factors c-Maf, NFIL3, and IRF4, suggesting incomplete differentiation (Supplementary Fig. 5a). Thus, treatment with αGalCer/CD1d-NPs, and to a much lesser extent αGalCer lipid alone, triggers the formation of LiNKTR1 cells at the expense of LiNKT cells.

**Table 2:** LiNKT cell clusters as identified by scRNAseq.

Gene	Cluster1	Cluster2	Cluster3	Cluster4	Cluster5
Zbtb16	+	+	+	+	++
Tbx21	+	+	−	+	+
Gata3	+	+	+	+	+
Rorc	−	−	+	−	−
Foxp3	−	−	−	−	−
Maf	+	+	++	+	+++
Nfil3	+	−	+	+	+
Bcl6	−	−	−	−	−
Irf4	−	−	−	−	+
Lef1	+	+	+	+	+
Cd24a	−	−	−	−	−
Cd44	+	+	++	+	++
Klrb1b	−	−	−	+	−
Klrb1c	+	+	−	+	−
Ifng	++	+	−	++	++
Il2	−	−	−	−	−
Il4	−	+	−	−	+
Il17a	−	−	−	−	−
Il10	−	−	−	−	++
Il21	−	−	−	−	+
Il2rb	++	++	+	++	++
Mirlet7b	−	−	−	−	−
Zfp683	−	−	−	−	−
Il17rb	−	−	+	−	−
Tnfsf11	−	−	+	−	−
Tnfrsf4	−	−	−	−	++
Il7r	++	+	+++	−	−
Tgfbr2	+	+	+	+	+
Lag3	−	−	+	+	+++
Ctla4	−	−	−	−	++
Slamf6	+	+	+	−	+
Itga4	−	−	−	++	++
Itgae	+	−	+	−	−
Pdcd1	−	−	+	−	+
Tigit	−	−	−	−	−
Ccr6	−	−	+	−	−
Ccr9	−	+	+	−	−
Cxcr5	−	−	−	−	−
Icos	+	+	++	+	++
Nrp1	−	−	+	−	+
Izumo1r	+	+	−	−	++
Cd27	+	+	+	+	++

Normalized gene expression values from 0–3.8: <0.1:–; 0.1–1:+;1-2:++;>2:+++.

Mass cytometry-based measurements of the presence of the LiNKTR1 cell cluster after 1, 3, 5, 7, and 10 doses of αGalCer/CD1d-NPs indicated that a minimum of three doses were required to generate the LiNKTR1 cell pool. However, the levels of expression of several LiNKTR1 markers (upregulation or downregulation) did not reach the 10-dose levels until after 5–7 doses (Supplementary Fig. 5b). scRNAseq studies of LiNKT cells isolated 14 and 35 days after treatment withdrawal revealed a significant reduction in the liver LiNKTR1 cell content of the treated mice to ~3% and 1%, respectively, along with an increase in the size of the LiNKT cell cluster #4, without significant changes in the size of clusters #1-#3 (Supplementary Fig. 5c). Together, these data suggest that LiNKTR1 cells either exit the liver (i.e., are not liver-resident), or have a short survival span.

### Human LiNKT cells are transcriptionally similar to their mouse counterparts

**Fig. 5:**
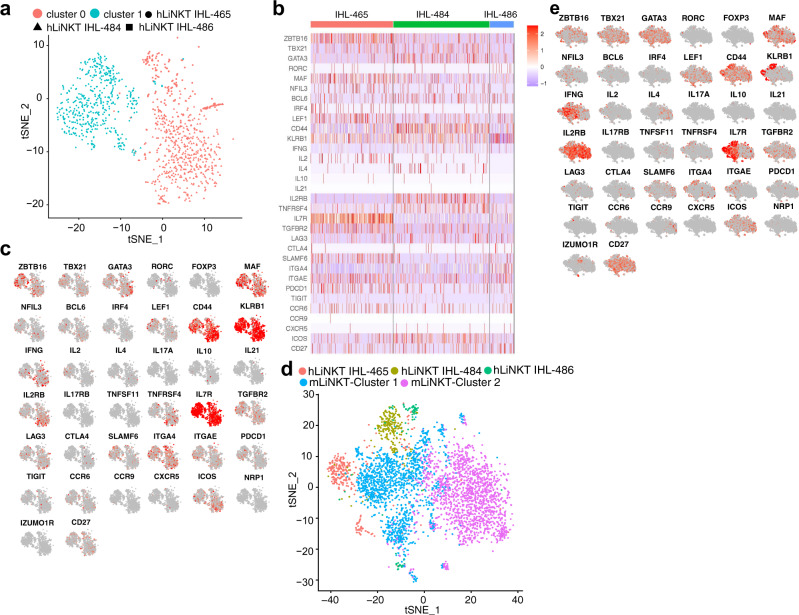
Single-cell RNAseq for human LiNKT cells. **a** tSNE plots from hLiNKT cells sorted from three different human liver explants. Each sample is labeled with a different symbol. The clusters the samples belong to are identified with different colors. **b** Heatmaps comparing expression levels for the genes from Suppl. Table 1 in the 3 hLiNKT cell samples. **c** Feature maps showing the cell cluster location of gene expression for the genes from Table 1. **d**, **e** Integrated tSNE plots (**d**) and feature maps (**e**) of mLiNKT clusters #1 and #2 and the three hLiNKT samples together.

We sorted αGalCer/CD1d tetramer+ LiNKT cells from intra-hepatic lymphocyte suspensions (IHLs) obtained from four liver explants of patients undergoing liver transplantation due to various conditions. We were able to obtain a sufficient number of LiNKT cells from three samples to proceed with scRNAseq (Fig. 5 and Supplementary Fig. 6). The cells from the three donors separated into two major clusters (Fig. 5a), with gene expression profiles remarkably similar to those of the prevalent mouse Zbtb16^+^Tbx21^+^Gata3^+^Maf^+^Rorc^–^ subset, particularly the mouse cluster #1 (Fig. 5b–e). Although there were clear inter-individual differences among the three hLiNKT samples that were studied and thus were not identical, they shared a similar expression profile for the 41 iNKT-relevant markers shown on Table 1, with the mouse LiNKT cluster #1 (Fig. 5e).

### LiNKTR1 cell-derived cytokines versus therapeutic activity

**Fig. 6:**
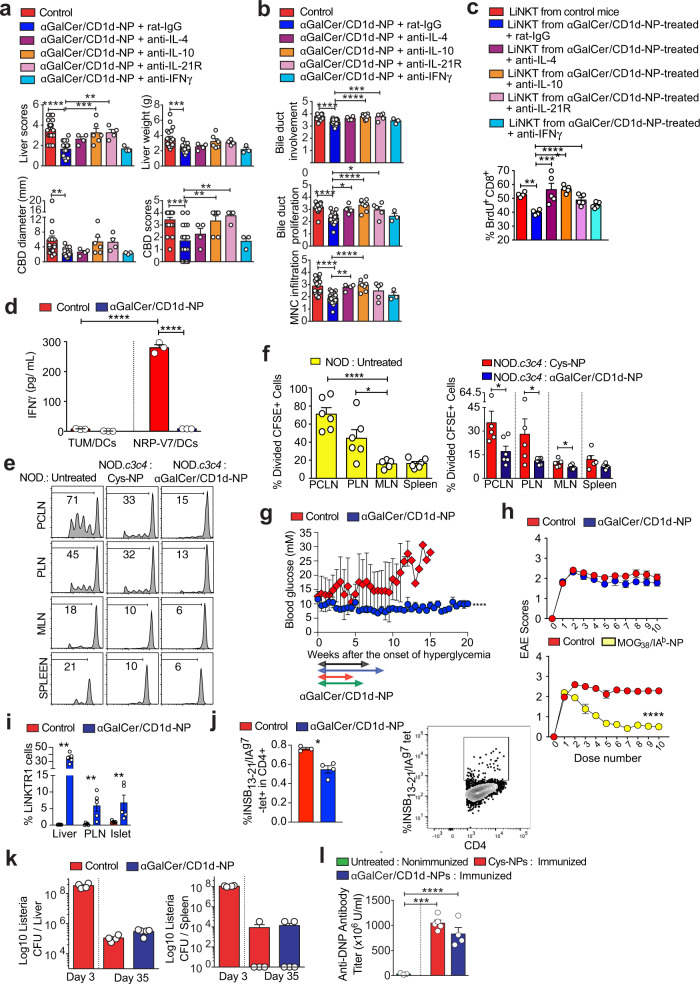
LiNKTR1 cytokine-mediated suppression of local and proximal APCs and diabetogenic autoimmunity without blunting normal immune responses. **a**, **b** Macroscopic (**a**) and microscopic (**b**) PBC scores of control NOD.*c3c4* mice and NOD.*c3c4* mice treated with αGalCer/CD1d-NPs along with rat-IgG or blocking mAbs against mouse IL-4, IL-10, IL-21Rα or IFNγ. In **a**, data correspond to *n* = 19 control mice (1 untreated and 18 Cys-NP-treated mice, pooled; 6 experiments) and *n* = 20 (6 experiments), 4, 6 (2 experiments), 4 and 3 mice treated with NPs and mAbs (from left to right). In **b**, data correspond to *n* = 21 control mice (1 untreated and 18 Cys-NP-treated mice, pooled; 6 experiments) and *n* = 23 (6 experiments), 4, 8 (3 experiments), 5 and 3 mice treated with NPs and mAbs (from left to right). All data were compared to the pMHCII-NP-treated group. **c** BrdU incorporation by splenic 8.3-CD8 + T cells in response to NRP-V7 peptide-pulsed liver KCs from NOD.*c3c4* mice, in the absence or presence of rat-IgG, or blocking mAbs against mouse IL-4, IL-10, IL-21Rα or IFNγ, and LiNTKs from control (Cys-NP-treated) or αGalCer/CD1d-NP-treated NOD.*c3c4* mice. Data correspond to *n* = 4, 4, 5, 5, 5, and 5/group. **d** IFNγ secretion by 8.3-CD8 + T cells from 8.3-NOD.*G6pc2*^–/–^.*Tcra*^–/–^ mice upon stimulation with NRP-V7- or TUM-peptide-pulsed CD11b + APCs isolated from the PCLNs of control NOD mice (treated with Cys-NPs (*n* = 3) or αGalCer/CD1d-NP-treated NOD mice (*n* = 3). **e**, **f** In vivo proliferation of naïve CFSE + 8.3-CD8 + T cells from 8.3-NOD.*G6pc2*^–/–^.*Tcra*^–/–^ donors in the PCLN, PLN, MLN and spleen of control NOD (*n* = 5–6/organ; top in **f**) and control (*n* = 5, treated with Cys-NPs) or αGalCer/CD1d-NP-treated NOD.*c3c4* mice (*n* = 6; bottom in **f**). **e** shows representative plots. **g** Evolution of blood glucose levels of acutely diabetic mice (>11 mM) in response to vehicle (*n* = 5) vs. αGalCer/CD1d-NP treatment (*n* = 4). Horizontal arrows show the duration of treatment with αGalCer/CD1d-NPs. **h** EAE scores (top) and body weight changes (bottom) of B6 mice with EAE in response to Cys-NP- (*n* = 9), αGalCer/CD1d-NP- (*n* = 10) or MOG_38-49_/IA^b^-NP (*n* = 6) treatment. Data correspond to two independent experiments. **i** Average percentages of LiNKTR1 cells (cluster #5) in liver, PLNs and pancreatic islets as determined via mass cytometry. Data correspond to 5 mice/organ. **j** Left: average percentages of InsB_13-21_/I-A^g7^ tetramer+ cells in islet-associated CD4 + T cells of NOD mice treated with αGalCer/CD1d-NP (*n* = 4) or control NPs (*n* = 3); Right: representative FACS profile. **k** Bacterial load of *L. monocytogenes* in the spleen and liver 3, and 35 days after infection (*n*  =  4, 4, and 5; left to right). **l** Serum anti-DNP antibody titers upon KLH–DNP immunization (*n*  =  3, 5 and 5; left to right). Data correspond to means ± SEM. *P* values were calculated using one-way ANOVA with Dunnett’s post hoc correction (**a**–**c**, **f**, **l**), one tail Mann–Whitney *U* (**d**, **i**, **j**, **k**), or two-way ANOVA (**g**, **h**). *P < 0.05; **P < 0.01; ***P < 0.001; ****P < 0.0001.

Systemic mAb-based IL-10 and IL-21 blockade significantly reduced the therapeutic activity of αGalCer/CD1d-NPs in NOD.*c3c4* mice (Fig. 6a, b). Whereas IL-4 blockade had a marginal effect, IFNγ blockade was inconsequential (Fig. 6a, b). In agreement with this, blocking mAbs against IL-4, IL-10, and IL-21Rα, but not IFNγ, inhibited the ability of LiNKT cells from αGalCer/CD1d-NP-treated NOD.*c3c4* mice to suppress the activation of splenic 8.3-CD8 + T cells by NRP-V7 peptide-pulsed liver KCs from NOD.*c3c4* mice (Fig. 6c). Thus, the therapeutic activity of αGalCer/CD1d-NPs against liver autoimmunity is largely IL-10 and IL-21-dependent, cytokines which also played a key role in autoreactive TR1-mediated suppression of inflammation^22–24^.

### In vivo suppression of local and liver-proximal APCs by LiNKTR1 cells

The above observations suggested that LiNKTR1 cells, as is the case for TR1-like CD4 + T cells, might suppress liver autoimmunity by both inhibiting the pro-inflammatory properties of local and/or proximal APC types as well as their ability to trigger the recruitment and/or activation of liver autoreactive T cells. LPS-challenged CD11b + cells from the PCLNs (liver-draining) of αGalCer/CD1d-NP-treated NOD.*c3c4* mice secreted significantly lower levels of a broad range of pro-inflammatory cytokines and chemokines (*n* = 24/29) than those isolated from control animals (Supplementary Fig. 7a, b). Likewise, KCs isolated from αGalCer/CD1d-NP-treated mice also secreted significantly lower levels of 19 of these 29 mediators (Supplementary Fig. 7c, d). No such differences were seen for CD11b + cells isolated from the mesenteric LNs (MLNs, non-liver-draining) of both types of mice (Supplementary Fig. 7e). Thus, treatment of NOD.*c3c4* mice with αGalCer/CD1d-NPs downregulates the pro-inflammatory capacity of both liver KCs and liver draining LN CD11b + cells.

Furthermore, whereas the PCLN-derived CD11b + DCs from untreated mice could readily trigger the activation of diabetogenic 8.3-CD8 + T cells ex vivo, upon pulsing with cognate (but not non-cognate) peptide, those isolated from αGalCer/CD1d-NP-treated mice were unable to do so (Fig. 6d). In addition, αGalCer/CD1d-NP-treatment almost completely abrogated the in vivo proliferative activity of exogenous 8.3-CD8 + T cells in both the PCLNs and PLNs (upon recognition of endogenous beta-cell-derived IGRP_206-214_) of treated NOD.*c3c4* mice (Fig. 6e, f). Thus, the therapeutic effects of αGalCer/CD1d-NP treatment involve the suppression of autoantigen presentation to non-cognate autoreactive T cells in the liver- and pancreas-draining lymph nodes.

### Suppression of liver-proximal but not -distal extra-hepatic autoimmunity by LiNKTR1 cells

The finding that αGalCer/CD1d-NP-induced LiNKTR1 cells could suppress autoantigen presentation in both the PCLNs and PLNs suggested that these compounds might also be able to suppress pancreatic beta cell autoimmunity. Indeed αGalCer/CD1d-NPs could restore normoglycemia in spontaneously diabetic NOD mice as compared to vehicle-treated controls, and these mice maintained normoglycemia for as long as 15 additional weeks post treatment withdrawal (Fig. 6g). In contrast, αGalCer/CD1d-NPs had no obvious therapeutic activity in B6 mice with EAE, as compared to cognate TR1 cell-inducing pMOG_38-49_/IA^b^-NPs (Fig. 6h). Analysis of the mass cytometry profiles of PLN and pancreatic islet-derived αGalCer/CD1d tetramer+ cells revealed the presence of a small, but clearly detectable contingent of LiNKTR1 cells in αGalCer/CD1d-NP-treated (up to 11-14% of cells, respectively) as compared to untreated NOD mice, where such cells were undetectable (Fig. 6i). Interestingly, the islet associated CD4 + T cells of αGalCer/CD1d-NP-treated mice contained significantly reduced percentages of Insulin_13-21_/I-A^g7^-specific CD4 + T cells, a prevalent population of islet-associated CD4 + T cells in NOD mice (Fig. 6j). This suggested that αGalCer/CD1d-NP therapy suppresses diabetogenesis in part by impairing the recruitment and activation of autoreactive T cells.

### αGalCer/CD1d-NP treatment spares normal immunity

These therapeutic effects against liver autoimmunity did not compromise the ability of the hosts to clear a *L. monocytogenes* (LM) infection, which infects liver hepatocytes as well as liver and splenic phagocytes but does not normally cause chronic infections. LM-infected αGalCer/CD1d-NP-treated and control NOD.*c3c4* mice were similarly able to reduce liver and splenic LM load (Fig. 6k). Likewise, αGalCer/CD1d-NP-treated and control NOD.*c3c4* mice produced similar titers of anti-dinitrophenyl (DNP) antibodies upon immunization with DNP-keyhole limpet haemocyanin (KLH) (Fig. 6l).

### Cognate LiNKTR1-B cell interactions in situ

Spinning-disk confocal intravital microscopy of endogenous liver B cells in αGalCer/CD1d-NP-treated Cxcr6^GFP/+^ B6.*Ifng-ARE-Del*^*+/−*^ mice, revealed an increased frequency (but not lengthening of the dwell time of the interaction) of local LiNKTR1–B-cell interactions both as measured in the field of view as well as over time (Supplementary Fig. 8a, b). Subsequent in vitro experimentation indicated that the liver B cells from NOD.*c3c4* mice had higher agonistic activity on LiNKT cells than NOD liver B cells, as measured by upregulation of CD69 (Supplementary Fig. 8c). Such differences were particularly pronounced when using LiNKTR1 cells from αGalCer/CD1d-NP-treated NOD.*c3c4* mice as responders (Supplementary Fig. 8c). These interactions between liver B cells and LiNKTR1 cells also triggered LiNKTR1 cell proliferation (Supplementary Fig. 8d) and cytokine secretion, particularly IL-21 (Supplementary Fig. 8e). B cell-induced LiNKTR1 activation, expansion and cytokine secretion required cognate interactions because they could be blocked with a CD1d-specific mAb (Supplementary Fig. 8c–e).

### LiNKTR1-driven Breg cell formation

Since pMHCII-NP-induced TR1-like CD4 + T cells trigger the formation of IL-10-producing Breg cells in an IL-21-dependent manner^22,23^, the above observations raised the possibility that LiNKTR1 cells might also be able to re-program conventional B cells into IL-10-producing Breg cells.

**Fig. 7:**
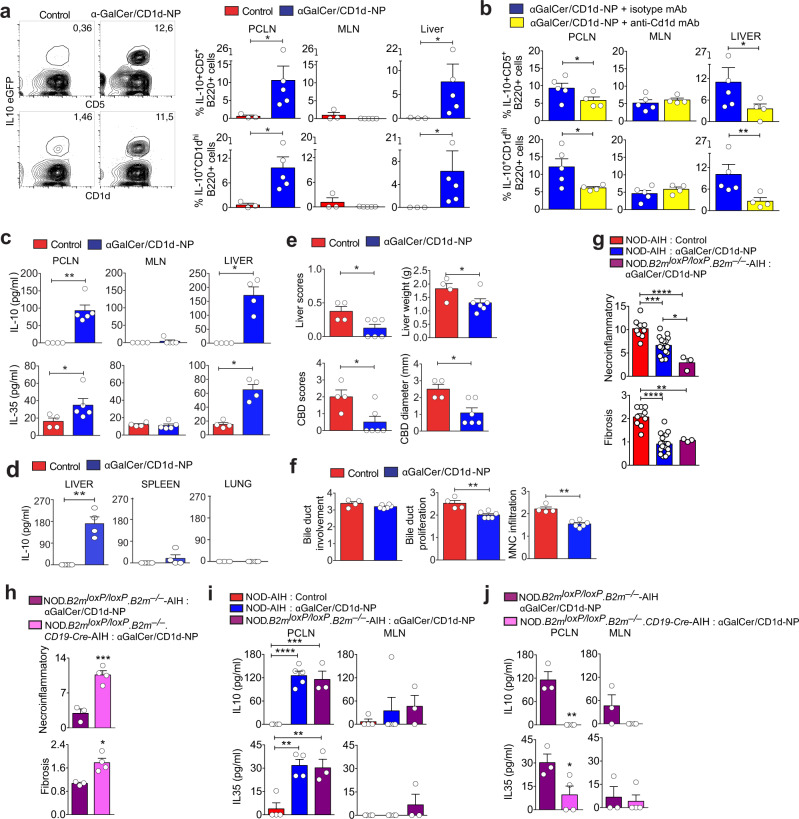
Local and proximal LiNKTR1-driven Breg cell formation via cognate interactions. **a**, **b** Conversion of adoptively transferred eGFP– B cells from NOD.*Il10-eGFP* donors into eGFP^+^CD5^+^CD1d^hi^ Bregs in the liver, PCLNs and MLNs of αGalCer/CD1d-NP- (*n* = 5) vs. Cys-NP-treated (*n* = 3) NOD.*c3c4* mice in the absence (**a**) or presence (**b**) of isotype (*n* = 4) or anti-CD1d mAb (*n* = 5). In **a**, Left panels show representative flow cytometric plots for liver B cells, and right panels show average percentages of eGFP^+^CD5^+^ and eGFP^+^CD1d^hi^ B cells within donor B cells. **c** Secretion of IL-10 and IL-35 by liver, PCLN and MLN B cells from Cys-NP- (*n* = 4) and αGalCer/CD1d-NP-treated (*n* = 5) NOD.*c3c4* mice, in response to an LPS challenge ex vivo. **d** Secretion of IL-10 by liver, splenic and lung B cells from Cys-NP- (*n* = 5) and αGalCer/CD1d-NP-treated (*n* = 4) NOD.*c3c4* mice, in response to an LPS challenge ex vivo. **e**, **f** Macroscopic (**e**) and microscopic (**f**) PBC scores in NOD.*c3c4.scid* hosts reconstituted with pooled splenocytes and PCLN cells from diseased NOD.*c3c4* donors upon transfusion with pooled liver and PCLN B cells from Cys-NP- (*n* = 4) or αGalCer/CD1d-NP-treated donors (*n* = 6). **g** Average microscopic liver scores in NOD or NOD.*B2m*^*loxP*^*.B2m*^*–/–*^ mice with hFTCD-induced AIH in response to treatment with Cys-NPs (*n* = 9, from 3 experiments) or αGalCer/CD1d-NPs (NOD mice: *n* = 15, from 4 experiments; NOD.*B2m*^*loxP*^*.B2m*^*–/–*^ mice: *n* = 3). **h** Average microscopic AIH scores in NOD.*B2m*^*loxP*^*.B2m*^*–/–*^ (*n* = 3), vs. NOD.*B2m*^*loxP*^*.B2m*^*–/–*^*/CD19-cre* mice (*n* = 4) in response to αGalCer/CD1d-NP treatment. **i** Secretion of IL-10 and IL-35 by PCLN- and MLN-derived B cells of the mice from **g** in response to an LPS challenge (*n* = 4, 4, and 3, respectively). **j** Secretion of IL-10 and IL-35 by PCLN- and MLN-derived B cells of the mice from **h** in response to an LPS challenge (*n* = 3 and 4, respectively). Data correspond to mean ± SEM. *P* values were calculated using one tail Mann–Whitney *U* (**a**–**f**, **h**, **j**) or one-way ANOVA with Tukey’s post hoc correction (**g**, **i**). *P < 0.05; **P < 0.01; ***P < 0.001; ****P < 0.0001.

We thus ascertained the ability of αGalCer/CD1d-NP-treated and control mice to support the differentiation of non-Breg cells from NOD.*Il10-eGFP* reporter mice (IL-10/eGFP– B cells) into CD1d^high^/eGFP+ and CD5 + /eGFP+ progeny in vivo. As shown in Fig. 7a, there was a clear formation of Breg cells in the liver and PCLNs, but not MLNs, of αGalCer/CD1d-NP-treated hosts, as compared to control hosts. Treatment of the hosts with anti-CD1d mAb abrogated LiNKTR1-driven Breg cell formation (Fig. 7b). In agreement with these data, the liver and the PCLN, but not the MLN, splenic or lung B cells from αGalCer/CD1d-NP-treated NOD.*c3c4* mice produced both IL-10 and IL-35 (major Breg cytokines^38,39^) as compared to those isolated from control NOD.*c3c4* mice (Fig. 7c, d). Furthermore, transfer of purified B cells pooled from the liver and PCLNs of αGalCer/CD1d-NP-treated NOD.*c3c4* mice (Supplementary Fig. 7f) suppressed the transfer of disease into NOD.*scid.c3c4* hosts reconstituted with splenocytes from diseased NOD.*c3c4* donors, as compared to those isolated from control-NP-treated donors (Fig. 7e, f). Thus, αGalCer/CD1d-NP-induced LiNKTR1 cells can trigger Breg cell formation via cognate interactions with B cells, and LiNKTR1-induced Breg cells, like LiNKTR1 cells, have independent immunoregulatory properties in vivo.

### Cognate LiNKTR1–B-cell interactions vs. Breg cell formation

We next used a genetic tool to investigate whether LiNKTR1-induced Breg formation requires cognate interactions between LiNKTR1 cells and B cells. We first induced AIH in NOD and CD19-Cre-transgenic NOD.*B2m*^*–/–*^ mice homozygous for a conditional b2m^loxP^ transgene (carrying CD1d-defficient B cells) (NOD.*B2m*^*–/–*^.*B2m*^*LoxP/LoxP*^.*CD19-Cre*) with the Ad-hFTCD adenovirus used in Fig. 1j, k. In agreement with the results of these earlier experiments, αGalCer/CD1d-NP therapy suppressed both necroinflammation and fibrosis in hFTCD-induced AIH in both NOD mice as well as CD19-Cre-negative NOD.*B2m*^*–/–*^*/B2m*^*loxP*^ mice (Fig. 7g). Notably, introduction of a CD19-specific β2 m deficiency into these mice significantly compromised the therapeutic activity of αGalCer/CD1d-NPs in this model (Fig. 7h), and this was associated with significant reductions in the ability of PCLN-derived B cells to produce IL-10 and IL-35 in response to LPS challenge ex vivo (Fig. 7i, j). Thus, the therapeutic activity of αGalCer/CD1d-NPs and their ability to trigger local Breg cell formation requires MHC class I expression in B cells, demonstrating a role for cognate iNKTR1-B-cell interactions in Breg cell formation.

## Discussion

We have shown that liver-resident αGalCer/CD1d-binding iNKT cells (LiNKT) comprise a large population of Tbx21^+^Gata3^+^Maf^low^Rorc^–^ cells that, although expressing an iNKT1-like cytokine profile, display a unique transcriptional signature and exhibit significant transcriptional, phenotypic and functional plasticity. Intravenous delivery of NPs displaying αGalCer/CD1d complexes triggered the differentiation of these LiNKT cells into a novel, immunoregulatory IL-10/IL-21-producing, Maf^high^ LiNKT subset that is distinct from all other iNKT cell subsets described to date, including iNKT10 (lacking *Zbtb16* expression^4^), and expresses a TR1-like transcriptional signature. These LiNKTR1 cells promote the conversion of local and proximal B cells into IL-10/IL-35-producing Breg cells via cognate, CD1d-restricted interactions and, as a result, suppress antigen-presentation to conventional autoreactive T cells in both the liver and liver- and pancreas-draining lymph nodes. Both LiNKTR1 and B cells isolated from the livers of treated mice could independently protect hosts from adoptive transfer of liver autoimmunity, and systemic αGalCer/CD1d-NP treatment reverted established liver autoimmunity and blunted the progression of beta cell destruction in acutely diabetic mice, without compromising normal immunity or blunting liver-distal autoimmunity.

We found that the LiNKT cells of untreated NOD, C57BL/6 and NOD.*c3c4* mice express a unique transcriptome and that they could not be classified into known iNKT cell subsets^1,4,5,9,10,31–37,40,41^. Although not identical, the LiNKT cells of all three genetic backgrounds shared key transcriptional differences with known iNKT cell subsets indicating that specification of their transcriptional profile is defined by the organ reservoir (i.e. liver) rather than by the disease status or genetic background of the mice, supporting local tissue specification. Single cell RNAseq of LiNKTs indicated the presence of two major LiNKT cell clusters conforming to the unique transcriptional profile of LiNKTs identified via bulk RNAseq, and a smaller cluster with an iNKT17-like transcription factor expression profile. Thus, most LiNKT cells belong to a bona fide novel subset, rather than a heterogeneous mixture of different thymic-derived iNKT cell subsets. For example, these two major LiNKT cell clusters co-expressed the iNKT1, iNKT2, iNKT17 transcription factor (TF)-coding genes *Tbx21*, *Gata3* and *Maf*, respectively, along with *Bcl6* and *Irf4*, involved in the lineage specification of T-Follicular helper cells^42^, and no or very low levels of the iNKT17 transcription factor gene, *Rorc*. In addition, these LiNKT cells expressed high levels of the iNKT1 cytokine gene *Ifng*, but very low or no levels of the cytokine genes *Il2*, *Il4, Il10*, *Il17a*, or *Il21* (Tables 1 and 2). These features were paralleled by similar differences in the levels of marker expression and cytokine secretion.

Remarkably, αGalCer/CD1d-NP therapy triggered the formation of yet another novel subset of LiNKT cells that shares many of the unique features of the two prevalent clusters of LiNKT cells, including high levels of *Zbtb16* expression (encoding PLZF), but massively upregulates the expression of *Maf* as well as genes coding for the co-inhibitory receptors Lag-3, Ctla-4, PD-1, and Tigit, the co-stimulator OX-40 (CD134), the NK co-receptor Slaf6, the lymphocyte homing receptor integrin CD49d, and the Folate Receptor 4 (Fr4), among others. Most of these transcriptional changes were mirrored by the corresponding upregulation of protein expression, as measured by conventional flow cytometry and multi-dimensional mass cytometry. Importantly, these re-programmed cells secrete high levels of the immunoregulatory cytokines IL-10 and IL-21, which are not expressed in the LiNKT cells of untreated mice (Tables 1 and 2). Although human LiNKT cells share key transcriptional features with their mouse counterparts, we do not know if they can also be re-programmed into LiNKTR1 cells. Unfortunately, this cannot be tested in vitro, as these compounds (as is also the case for the pMHCII-NP compounds that trigger cognate TR1 cell formation) can only re-program CD4+ and LiNKT cells in vivo. The reasons behind this remain unclear, but one possibility is that sustained in vitro culture of responder T cells with cognate MHC-NPs somehow overrides in vivo re-programming effects that might be driven by relatively short, but repetitive exposures of cognate T cells to these compounds in vivo, based on their extremely fast pharmacokinetic behavior^43^.

Co-expression of IL-10 and IL-21, along with the transcription factors c-Maf, T-bet, Bcl6, Irf4, and Nfil3 and the checkpoint inhibitors Lag-3, PD-1, Ctla-4, and Tigit, among others, parallels the upregulation of the same genes at similar levels by the pMHCII-NP-induced (IL-10/IL-21-expressing) TR1-like cells described by us recently^22^, suggesting that the precursors of pMHCII-NP-induced TR1 and αGalCer/CD1d-induced LiNKTR1 cells interpret sustained ligation of their TCRs by the cognate nanomedicines similarly^21^. Since both LiNKTR1 and CD4 + TR1 cells use IL-10 and IL-21 and similar mechanisms to suppress autoimmunity, and since both cell types have intrinsic disease-suppressing properties, our data suggest the possibility that simultaneous treatment with both compounds might have superior (additive or synergistic) therapeutic effects.

Importantly, neither the splenic nor the lung iNKTs acquired the IL-10/IL-21 secretory competence of LiNKTR1 cells in response to αGalCer/CD1d-NP therapy. Since iNKT cells residing in organs other than the liver, including the lung and spleen, do not access the vasculature, it is reasonable to suspect that this is because αGalCer/CD1d-NPs can only readily engage LiNKTs. Alternatively, LiNKT cells are uniquely poised to differentiate into LiNKTR1 cells in response to sustained TCR ligation. Whether extra-hepatic iNKT cell subsets other than those residing in the splenic and lung, such as undifferentiated stage 0 thymic iNKTs^10,44^, can also be re-programmed into LiNKTR1-like cells remains to be determined. However, the inability of αGalCer/CD1d-NPs to blunt liver-distal autoimmunity (EAE) or normal immune responses (LM infection in both liver and spleen, as well as antibody responses to a nominal antigen vaccine), argues against this scenario. We note that LiNKTR1 cells retain the ability to secrete IFNγ upon TCR ligation, which plays a critical role in liver iNKT-mediated protection from LM and other liver pathogens^5^. This is also the case for the TR1 CD4 + T cells induced by pMHCII-NP therapy. However, IFNγ (unlike IL-10 and IL-21) blockade neither impaired nor enhanced the anti-diabetogenic or anti-hepatogenic properties of pMHCII-NP-induced TR1 cells^22,23^ or the anti-hepatogenic properties of αGalCer/CD1d-NP-induced LiNKTR1 cells (this manuscript). Since IFNγ-deficient NOD mice develop diabetes essentially like their wild-type counterparts, yet IFNγ plays a critical role in CFA-mediated protection from T1D^45^, it is likely that this cytokine can have both pro- and anti-inflammatory properties in a context-dependent manner. In the case of LiNKTR1 and TR1 cells, the potential pro-inflammatory properties of IFNγ might be offset by the anti-inflammatory properties of IL-10 and IL-21.

Whatever the case might be, the regional regulatory specificity of LiNKTR1 cells further suggests that these cells largely reside in the liver and/or liver-associated lymphoid tissues, unlike the case for autoantigen specific TR1 cells, which can traffic to sites of inflammation, even in the absence of cognate autoantigen expression^24^. On the other hand, the size of the intra-hepatic LiNKTR1 cell subset decreases rapidly after treatment withdrawal, suggesting that these cells exit the liver shortly upon induction (i.e., are not strictly liver-resident) or are short-lived. The presence of LiNKTR1-like cells in the pancreatic islets and pancreatic lymph nodes (PLNs) of the αGalCer/CD1d-NP-treated mice supports the former possibility, although it does not exclude the latter. The ability of LiNKTR1 cells to suppress regional autoimmunity, such as T1D, may thus be mediated by these islet/PLN-associated LiNKTR1 cells and/or by the Breg cells that arise downstream of LiNKTR1 cell re-programming, by suppressing autoantigen presentation in the PLNs (which also drain the liver). Long-term persistence of the anti-diabetogenic effects of αGalCer/CD1d-NP therapy in diabetic mice might in fact be sustained by these LiNKTR1-induced Breg cells. In this regard, it is worth noting that the PLN-associated Breg cells arising in pMHCII-NP-treated NOD mice have independent anti-diabetogenic properties that synergize with those of their antigen-specific TR1 counterparts^22^. Since the efficiencies of LiNKTR1 and TR1 cell-driven Breg cell formation, as measured in NP-treated hosts engrafted with B cells from NOD.*Il10*^tm1Flv^ donors, and the magnitude of IL-10 secretion by the PLN-associated B cells of pMHCII-NP- and αGalCer/CD1d-NP-treated mice were similar, it is likely that the anti-diabetogenic activity of αGalCer/CD1d-NPs is, in part, mediated by these Breg cells.

Given the upregulation of c-Maf, a strong positive regulator of IL-10 and IL-21 expression, by both LiNKTR1 and TR1 CD4 + T cells, it is reasonable to suspect that this transcription factor plays a critical role in shaping the regulatory phenotype of LiNKTR1 cells. c-Maf is responsible for the activation or suppression of specific cytokine-encoding genes in CD4 + T cells, including Treg cells^46–49^, as well as iNKT cells^50^, in a cell-context-dependent manner^51^. As expected based on the fact that c-Maf expression in Th2 cells is critical for IL-4 expression^46,52^, the LiNKTR1 cells described herein also upregulate *Il4*, in addition to *Il10* and *Il21*. In contrast, although c-Maf has been implicated in Th17, iNKT17 and, more recently, γδT17 cell differentiation (ref. ^53^ as well as *Il17a/f* expression by inducing the expression of *Rorc*^50,54^), LiNKTR1 cells express neither *Rorc* nor *Il17a/f* despite expressing *Maf*. Furthermore, whereas c-Maf suppresses the expression of *Ifng* by antagonizing *Ifng*-inducing *Tcf7* in γδT17 cells^53^, LiNKTR1 cells co-express both *c-Maf* and *Ifng*. It is therefore conceivable that c-Maf expression is necessary but not sufficient to elicit the transcriptional program seen in LiNKTR1 cells. It is also interesting to note that LiNKTR1 cells co-express the Th2 transcription factor Gata-3. In Th2 cells, Gata-3 triggers chromatin changes at the *Il10* locus and these changes are necessary, albeit not sufficient for IL-10 expression^55^. On the other hand, in Th1 and Th17 cells (lacking Gata-3 expression), other factors, such as E4bp4, are responsible for inducing the epigenetic changes at *Il10* that enable IL-10 expression upon chronic TCR ligation^56^. Notably, both LiNKTR1 and pMHCII-NP-induced TR1-like cells express E4bp4. Although defining the specific role that each of these and other transcription factors play in LiNKTR1 specification in response to αGalCer/CD1d-NPs will require additional experimentation, it seems very likely that the unique transcriptional and/or epigenetic footprint of LiNKT cells is required for LiNKTR1 cell re-programming. An additional aspect that we are seeking to clarify is whether the LiNKTR1 subset arises from either one or both of the two major subsets of LiNKT cells that we find in untreated mice.

Systemic delivery of αGalCer/CD1d-NPs was able to comprehensively blunt spontaneous and experimental liver autoimmunity in various genetic backgrounds, consistent with a robust immunoregulatory activity of the locally re-programmed LiNKTR1 cells that is dissociated from disease type (PBC vs. AIH) and genetic background (C57BL/6 vs. NOD.*c3c4*). It is worth noting that administration of soluble αGalCer lipid did not have any therapeutic activity in these models, consistent with the fact that MHC-based nanomedicines operate via direct and sustained ligation of antigen receptors on cognate T cells^21^. Remarkably, αGalCer/CD1d-NPs were also able to rapidly restore and stably maintain normoglycemia in spontaneously diabetic NOD mice for many weeks post-treatment withdrawal, despite the fact that these mice do not have liver inflammation. This ability of LiNKTR1 cells to suppress pancreatic autoimmunity is due to two facts, (i) that the pancreatic and portal/celiac lymph nodes simultaneously drain both the liver and the pancreas; and (ii) that the re-programmed LiNKTR1 cells (or the Breg cells arising downstream of LiNKTR1 cells) can suppress antigen presentation and autoreactive T-cell activation at both sites. Specifically, IGRP_206-214_-specific 8.3-CD8 + T cells (recognizing an epitope from an autoantigen that is exclusively expressed in pancreatic beta cells) proliferate in both lymph node types (but not elsewhere^57^) and αGalCer/CD1d-NP therapy effectively blunted this in vivo proliferative response. These observations are consistent with two additional observations: (i) LiNKTR1cells from αGalCer/CD1d-NP-treated mice suppressed the ability of normal liver KCs and liver-draining CD11b + APCs to trigger 8.3-CD8 + T-cell activation in vitro; and (ii) KC and lymph node-derived CD11b + cells from αGalCer/CD1d-NP-treated mice displayed impaired antigen-presentation and pro-inflammatory capabilities ex vivo.

Although purified LiNKTR1 cells could effectively transfer disease protection to hosts reconstituted with pathogenic effectors, their immunoregulatory activity in response to αGalCer/CD1d-NP therapy was amplified in vivo by the local and regional (but not liver-distal) formation of IL-10 and IL-35-producing Breg cells. In vivo, the liver-resident LiNKTR1 cells were observed to directly interact with B cells. In vitro, liver-associated B cells triggered LiNKTR1 cell activation, proliferation and cytokine secretion, particularly of IL-21, in a CD1d-dependent manner. Two specific observations demonstrated that these LiNKTR1-B-cell interactions promoted Breg cell formation: (i) when transferred into αGalCer/CD1d-NP-treated mice, conventional B cells acquired the ability to express IL-10 in vivo; and (ii) the liver- and liver draining lymph node-derived B cells from αGalCer/CD1d-NP-treated mice contained IL-10 and IL-35-secreting B cells. Indeed, B cells purified from the livers and liver-draining lymph nodes of treated animals could transfer disease protection to hosts engrafted with pathogenic effectors, thus indicating an active role in disease suppression. Since selective deletion of CD1d in B cells in mice with PBC significantly impaired the therapeutic activity of αGalCer/CD1d-NP treatment, it is likely that these LiNKT-B-cell interactions play critical roles not only in Breg cell formation but also in LiNKTR1 cell activation and homeostasis. Indeed, it has been shown that Cd1d^high^ T2-MZP Breg cells can present endogenous lipids to iNKT1 cells, triggering IFNγ secretion and the suppression of Th1 and Th17 adaptive immune responses in an IL-10-independent manner^37^. In addition, iNKT-FH cells can provide cognate T-cell help to follicular B cells^34^, and marginal zone B cells can trigger iNKT cell activation^58,59^.

Since TR1 cell-driven Breg cell formation is IL-21-dependent^22,23^ and LiNKTR1 cells produce significant amounts of IL-21, it is likely that LiNKTR1 cell-driven Breg cell formation is also mediated via IL-21. This is consistent with the effects of IL-21R blockade on the outcome of pMHCII-NP therapy in various autoimmune disease models where we have documented TR1 cell-driven, IL-21-dependent Breg cell formation. For example, IL-21R blockade in NOD mice treated with T1D-relevant pMHC class II-NPs abrogated the suppression of autoantigen cross-presentation to diabetogenic CD8 + T cells in the pancreatic lymph nodes, the anti-encephalitogenic activity of EAE-relevant pMHCII-NPs in HLA-DR4-transgenic B6 mice^22^, and the anti-hepatogenic activity of liver autoimmune disease-relevant pMHCII-NPs in NOD.*c3c4* mice^23^. We further note that IL-21 has also been described to contribute to Breg cell formation and Breg cell-dependent suppression of disease progression in EAE^60^. Although these data seem to be at odds with the pro-diabetogenic effects of IL-21 in T1D^61,62^, IL-21 shares paradoxical disease-promoting and disease-suppressing effects with other Breg-inducing cytokines with pleiotropic activity, where the outcome of cytokine signaling is context dependent^63^.

Whatever the specific role of IL-21 might be, this LiNKTR1 cell-driven Breg cell formation process involves CD1d-restricted interactions between the LiNKTR1 cells and B cells. However, whereas TR1-driven Breg cell formation in vivo is peptide-specific, LiNKTR1-driven Breg cell formation occurred in the absence of αGalCer pulsing, suggesting the involvement of endogenous glycolipids, possibly α-glycosylceramides (the principal endogenous ligands for iNKT cells in mammalian cells^64^). Clearly, expression of these lipid/CD1d complexes on B cells does not require the presence of liver inflammation and occurs in the steady-state since LiNKTR1 cells can readily trigger IL-10 expression in conventional B cells from healthy donors in vivo. However, it is possible that the local inflammatory microenvironment in these mice triggers the upregulation of endogenous glycolipids, such as the lysosomal glycosphingolipid iGb3^65^, a possibility supported by the observation that liver B cells from diseased NOD.*c3c4* mice have superior agonistic activity on LiNKTR1 cells than B cells from healthy donors. Additional evidence supporting the presence of endogenous lipid presentation to iNKT cells via CD1d on B cells is the observation that iNKT cells have been shown to promote the generation of alloreactive antibodies in liver transplantation^66^ or the expansion of CD1d^high^CD5 + B cells in vitro in a cell-contact-dependent and αGalCer-independent manner^67^.

In sum, the work described herein has unearthed the existence of a novel immunoregulatory iNKT cell subset that arises from a novel liver-resident precursor, with profound anti-inflammatory activity against liver and diabetogenic autoimmunity, in part by promoting the regional formation of Breg cells. Our observations thus demonstrate that at least certain tissue-resident iNKT cell subsets are plastic and can be re-programmed with lipid/CD1d-based nanomedicines to induce comprehensive disease-agnostic, organ-specific therapeutic activity. Delivery of these compounds via routes capable of accessing other tissue-resident iNKT cells might be able to treat other organ-specific inflammatory diseases.

## Methods

### Mice

NOD/LtJ (JAX# 001976), C57BL/6 (B6) (JAX# 000664), NOD.*c3c4* (JAX# 010971), and B6.*Cxcr6*^*Gfp*^ (JAX# 005693) mice were purchased from the Jackson Laboratory (Bar Harbor, ME). 8.3-NOD.*G6pc2*^*–/–*^.*Tcra*^*–/–*^ mice have been described^68^. B6.*Ifng-ARE-Del*^*−/−*^ mice were obtained from H. Young (NIH, Bethesda, MD)^26^. NOD.*c3c4*.*scid* mice were generated by backcrossing (NOD.*c3c4* x NOD.*scid*) F1 mice with NOD.*c3c4* mice for five generations, followed by intercrossing of mice heterozygous for the *scid* mutation and homozygous for the B6 chromosome 3 and 4 intervals from NOD.*c3c4* mice. (NODxB6.*Ifng-ARE-Del*^*–/–*^*)* F1 mice were generated by intercrossing B6.*Ifng-ARE-Del*^*−/−*^ and NOD/LtJ mice. B6.*IFNγ ARE-Del*^*+/–*^ mice were generated by intercrossing B6.*Ifng-ARE-Del*^*−/−*^ and B6 mice. B6.*Ifng-ARE-Del*^+/–^.*Cxcr6*^*GFP/+*^ mice were generated by intercrossing B6.*Ifng-ARE-Del*^*−/−*^ and B6.*Cxcr6*^*Gfp*^ mice. NOD.*Il10*^*tm1Flv*^ (Tiger) mice were obtained by backcrossing the *Il10*^*tm1Flv*^ allele from C57BL/6.*Il10*^*tm1Flv*^ mice (JAX# 008379) onto the NOD/Ltj background for 10 generations. NOD.*B2m*^*loxP/loxP*^/*B2m*^*–/–*^^69^ and NOD.*B2m*^*loxP/loxP*^/*B2m*^*–/–*^.*CD19-Cre* have been described^68,70^. All mice were housed in specific pathogen-free conditions and experimental and control animals were co-housed. Mice were euthanized via cervical dislocation. These studies were approved by the animal care committee of the Cumming School of Medicine at the University of Calgary (Health Sciences Animal Care Committee (HSACC)).

### Adenovirus

Replication-deficient adenoviruses expressing human formiminotransferase cyclodeaminase (Ad-hFTCD) were generated as described^23,71^. The viruses were amplified in Ad-293 T cells and purified using Adeno-X Maxi Purification Kit (Clontech). The viral titer was measured using the End-point Dilution Assay or Adeno-X Rapid Titer Kit (Clontech).

### Human samples

Human liver-associated iNKT cells were purified from white blood cell isolates obtained from whole liver explants collected at the time of liver transplantation under informed consent at the University of Alberta. Briefly, the liver explants were flushed for 20 min with PBS to remove red blood cells and other circulating cell types, followed by a 2L hard flush optimized to yield cell mononuclear recoveries from liver that are similar to those obtained after mechanical disruption. The collected cells were then stored in liquid nitrogen. The liver isolates studied herein were obtained from 4 patient samples: IHL-453 (61-year-old female with alcoholic liver disease and cirrhosis with past medical history of obesity and type 1 diabetes); IHL-465 (58-year-old female with non-alcoholic steatohepatitis, cirrhosis and an hepatocellular carcinoma, past medical history of hypertension and type 2 diabetes); IHL-484 (38-year-old male with Wilson’s disease); and IHL-486 (63-year-old male with resolved hepatitis C virus infection and hepatocellular carcinoma, with past medical history of dyslipidemia). The studies described herein were approved by the institutional ethic boards of both the University of Alberta (Health Research Ethics Board (HREB)) and the University of Calgary (Conjoint Health Research Ethics Board (CHREB)).

### Flow cytometry

Fluorochrome-conjugated antibodies specific for mouse CD5 (53-7.3), CD19 (1D3), CD27 (LG.3A10), CD69 (H1.2F3), CD49d (MFR4.B), CD122 (TM-β1), CD134 (OX-86), CD1d (1B1), Ctla-4 (UC10-4F10-11), Lag-3 (C9B7W), Icos (C398.4A), CD11b (M1/70), TCRβ (H57-597), B220 (RA36B2), and streptavidin–PerCP and streptavidin–PE were purchased from BD Biosciences (San Diego, CA). Fluorochrome-conjugated antibodies specific for mouse CD127 (A7R34) and F4/80 (BM8) were purchased from eBioscience (San Diego, CA). Anti-CD4 (RM4-5), -CD8 (53-6.7), and -PD1 (29 F.1A12) mAbs were purchased from BioLegend (San Diego, CA) and anti-Tigit (GIGD7) was purchased from Invitrogen. For intracellular staining of transcription factors, we first sorted αGalCer/CD1d tetramer+ cells (see below) and then stained the sorted cells for the various TFs using the FoxP3/Transcription Factor Staining Buffer Set (eBioscience/Thermo Fisher Scientific). Anti-T-bet (4B10) was from BioLegend. Anti-Plzf (R17-908), anti-Gata-3 (L50-823), and anti-Rorγt (Q31-378) were from BD Pharmingen, and anti-c-Maf (sym0F1) was from Invitrogen. Cells were preincubated with Fc block (BD Pharmingen) before antibody staining.

For tetramer staining, cells were stained with αGalCer/CD1d Tetramer (2.5 μg ml^−1^) in FACS buffer (0.05% sodium azide and 1% FBS in PBS) for 60 min at room temperature, and then with FITC-conjugated anti-mouse TCRβ (5 μg ml^−1^) for 30 min at 4 °C. After washing, cells were fixed (1% paraformaldehyde in PBS) and analyzed in BD CytoFLEX or LSRII flow cytometers. Analysis was performed using FlowJo v10 software (TreeStar).

### Mass cytometry

Liver mononuclear cells from αGalCer/CD1d-NP-treated and untreated NOD mice (*n* = 3 each) were incubated with anti-CD16/32 (10 µg/ml, BioLegend) for 10 minutes and αGalCer/CD1d-tetramer-APC (5 µg/ml, produced in house) for 30 min at room temperature. Surface markers were stained with anti-TCRβ-143Nd, anti-CD27-150Nd, anti-CD49d-151Eu, anti-CTLA4-154Sm, anti-PD1-159Tb, anti-ICOS-168Er, anti-LAG3-174Yb, anti-CD127-175Lu, anti-APC-176Yb (Fluidigm, San Francisco, CA), anti-TIGIT-152Sm, anti-SLAF6-156Gd, anti-CD122-169Tm, anti-TGFBR2-170Er, anti-FR4-171Yb (mAbs from ThermoFisher labeled in-house with a kit from Fluidigm), and anti-CD134-161Dy (mAb from BD Biosciences labeled in-house) for 30 minutes at 37 °C. After Cysplatin viability staining, cells were fixed with 1:1 mixture of FoxP3-Fixation/Permeabilization solution (ThermoFisher) and Cytofix/Cytoperm solution (BD Biosciences) for 30 minutes on ice. Transcription factors were stained with anti-IRF4-155Gd, anti-BCL6-163Dy, anti-GATA3-167Er (Fluidigm), anti-cMAF-141Pr, anti-FOXP3-145Nd (mAbs from ThermoFisher labeled in-house), anti-RORγt-147Sm (mAb from BD Biosciences labeled in-house), anti-PLZF-149Sm, anti-NFIL3 (mAb from R&D Systems, Minneapolis, MN labeled in-house), anti-TBX21-153Eu (mAb from BioLegend labeled in-house) for 30 min at 4 °C. After Intercalator staining for overnight at 4 °C, cells were acquired with a Helios Mass Cytometer (Fluidigm). FCS files were analyzed using FlowJo to select single alive iNKT cells (APC + TCRβ^int^), and then further analyzed with viSNE using Cytobank Premium (Cytobank, Santa Clara, CA) using the default settings.

### Spinning disc confocal intravital microscopy (SD-IVM)

A tail vein catheter was inserted into mice after anesthetization with 200 mg/kg ketamine and 10 mg/kg xylazine. Mouse body temperature was maintained at 37 °C with a heated stage. Image acquisition was performed using an Olympus IX81 inverted microscope, equipped with an Olympus focus drive and a motorized stage, and coupled to a confocal light path based on a modified Yokogawa CSU-10 head. Target cells were visualized using fluorescently stained antibodies. KCs and B cells were stained by i.v. injection of 2.5 μg anti–F4-80 (clone BM8) or 3.5 μg anti-CD19 (clone 1D3) fluorescent conjugated mAbs. Volocity v7 and Image J v1.44 software were used to drive the confocal microscope, for 3D rendering, acquisition, and analysis of images, as well as to track iNKT accumulation and interactions with KCs and B cells. We used 2 mm^2^ stitch images of the liver making use of the find object function and appropriate thresholding^72^.

### CD1d and pMHCII monomer and tetramer production

Empty mouse CD1d and pMHC class II (Insulin_13-21_/I-A^g7^) monomers were purified from supernatants of CHO-S cells (Invitrogen, San Diego, CA) transduced with lentiviruses encoding a monocistronic message in which β2m and CD1d or peptide-MHCβ and MHCα chains were separated by the ribosome skipping P2A sequence (the vector sequences were assembled and annotated using Geneious Prime). The CD1d monomers were engineered to encode a BirA site, a 6×His tag and a free Cys at the carboxyterminal end of the construct. The self-assembled CD1d complexes were purified by nickel chromatography. In pMHCII monomers, the peptide was tethered to the amino terminal end of the MHCβ chain via a flexible GS linker and the MHCα chains were engineered encode a BirA site, a 6xHis tag, a twin strep-tag and a free Cys at their carboxyterminal end. The secreted, self-assembled pMHC class II complexes were purified by sequential nickel and Strep-Tactin chromatography. The purified mCD1d and pMHCII monomers were used for coating onto NPs and/or processed for biotinylation and tetramer formation using Streptavidin-APC/PerCP/PE (from BD Biosciences or Agilent Technologies). To prepare αGalCer-loaded mCD1d tetramers, KRN 7000 was added into the biotinylated monomers in the presence of 0.05% PBST and incubated for 3 h at 37 °C followed by addition of Streptavidin-fluorochrome conjugates and an overnight incubation at 4 °C.

### Nanoparticle synthesis and mCD1d/pMHCII conjugation and αGalCer loading

Maleimide-functionalized, pegylated iron oxide NPs (PFM series) were produced in a single-step thermal decomposition in the absence of surfactants as described recently^21^. Briefly, 3 g Maleimide-PEG (2 kDa MW, Jenkem Tech USA) were melted in a 50 mL round bottom flask at 100^o^C and then mixed with 7 mL of benzyl ether and 2 mmol Fe(acac)_3_. The reaction was stirred for 1 h and heated to 260 °C with reflux for 2 hr. The mixture was cooled to room temperature and mixed with 30 mL water. Insoluble materials were removed by centrifugation at 2000×*g* for 30 min. The NPs were purified using magnetic (MACS) columns (Miltenyi Biotec, Auburn, CA) and stored in water at room temperature or 4 °C. The concentration of iron was determined spectrophotometrically at 410 nm in 2 N hydrochloric acid (HCl). To conjugate mCD1d or pMHCII onto PFM, mCD1d and pMHCII monomers, respectively, were mixed with NPs in 40 mM phosphate buffer, pH 6.0, containing 2 mM ethylenediaminetetraacetic acid (EDTA), 150 mM NaCl, and incubated overnight at room temperature. mCD1d and pMHCII-conjugated NPs were purified by magnetic separation and concentrated by ultrafiltration through Amicon Ultra-15 (100 kDa cut-off) and stored in PBS. To load αGalCer onto mCD1d-NPs, KRN 7000 (Cayman Chemical, Ann Arbor, MI), dissolved in DMSO, was added to the mCD1d-NP suspension at a molar ratio of 12:1, respectively in the presence of 0.05% PBST and incubated for 3 h at 37 °C followed by overnight at 4 °C. The αGalCer-loaded CD1d-NPs were subjected to magnetic purification and then sterilized by filtration through 0.2 μm filters and stored at 4 °C. The size and dispersity of unconjugated and mCD1d- or pMHC-conjugated NPs were assessed via transmission electron microscopy (TEM, Hitachi H7650) and dynamic light scattering (DLS, Zetasizer, Malvern, UK). Pegylated and CD1d/pMHC-NPs were also analyzed via 0.8% agarose gel electrophoresis and native- and denaturing 10% SDS-PAGE. To quantify mCD1d or pMHC valency, we measured the mCD1d and pMHC concentration in the mCD1d- and pMHCII-NP preps, respectively using the Bradford assay (Thermo Scientific).

### Autoimmune disease models and αGalCer/CD1d-NP therapy

Cohorts of NOD.*c3c4* (~15 weeks old, males and females) and (NODxB6.*Ifng-ARE-Del*^–/–^) F1 or *Cxcr6*^eGFP+/–^ B6.*Ifng-ARE-Del*^*+/–*^ mice (~10-week-old females) were left untreated or treated with 10–14 doses of 20 μg (pMHC weight) of αGalCer/CD1d-NPs, control Cys-NPs (the exact same amount of iron given with the pMHC-coated NPs) or 400 ng of αGalCer (the molar equivalent of αGalCer delivered by 20μg of αGalCer/CD1d-NPs) (i.v.). The first 10 doses of NPs were administered twice a week and any additional doses were given once a week.

AIH was induced in NOD mice by injecting 1 × 10^10^ PFU of a replication-defective adenovirus encoding the human AIH Type 2 target autoantigen FTCD (Ad-hFTCD) i.v.^27^. Four weeks later, cohorts of mice with established AIH were treated with 20 μg of αGalCer/CD1d-NPs or control Cys-NPs i.v. twice weekly for 5–6 week.

To induce EAE, female 8-week-old C57BL/6 mice were immunized with 200 μg of pMOG_36–55_ in CFA s.c. and received 300 ng of Pertussis toxin i.v. on days 0 and 3 relative to peptide immunization. Since these mice develop a synchronous non-remitting form of chronic EAE, all the mice were randomized into treatment (Cys-NPs, αGalCer/CD1d-NPs or pMOG_38-49_/IA^b^-NPs –20μg of pMHC/dose) when they reached a score of 1.5. Mice were scored and weighted on day 0 and then daily from day 7 to 10 after immunization, and scores were plotted on a 5-point scale as described^22^.

Experiments in diabetic mice involved following cohorts of 13-week-old female NOD/Ltj for diabetes development by measuring blood glucose levels with Accucheck Strips (Roche) twice a week. Mice displaying blood glucose measurements >11 mM were considered diabetic and treated twice weekly with 20 μg αGalCer/CD1d-NPs or control Cys-NPs until stably normoglycemic (defined as 8 consecutive measurements <11 mM) or until hyperglycemia was considered irreversible (3 measurements >25 mM). Animals were assessed daily for glycosuria (corresponding to >15 mM blood glucose) and given human insulin isophane (1IU per day) s.c. if positive.

### Histological scoring of liver autoimmune disease

Livers were fixed in 10% formalin for 2 days, embedded in paraffin, cut into 5 μm sections and stained with H&E or Picrosirius Red. The sections were scored by two independent investigators and reported as averages of the two. Briefly, ~0.5 cm^2^ sections from each of the four liver lobes from each mouse (right and left, median and caudal) and a minimum of 4 portal triads per lobe section (16 portal triads/mouse) were scored.

Liver disease scoring in NOD.*c3c4* mice involved macroscopic evaluation of cyst content (0–5, with 0.5 increments except in adoptive transfer studies where 0.25 increments were used), liver weight and CBD diameter (0–4), as well as microscopic evaluation of bile duct involvement (0–4, with 0.5 increments), bile duct proliferation (0–4, with 0.5 increments), and mononuclear cell infiltration (0–4, with 0.5 increments), essentially as described^23^.

Histopathologic scoring for PBC in the livers from B6.*Ifng-ARE-Del*^*+/–*^ was done as described^23,26^. Briefly, severity scores were obtained by scoring portal inflammation, lobular inflammation and granuloma formation from 0 to 4, and bile duct damage from 0 to 2. The extent of portal inflammation and bile duct damage were scored from 0-4 based on the ratio between affected vs unaffected area. The extent of lobular inflammation and granuloma formation were scored from 0 to 4 based on number of lesions per specimen. Inflammatory scores were obtained by adding the scores for both severity and lesion number. The severity of fibrosis was scored on a 0–6 scale as described^73^.

Histopathological scoring of AIH was done using the Ishak scale as above^73^, which assesses both fibrosis (0–6) as well as necroinflammatory sequelae, including interface hepatitis (0–4), confluent necrosis (0–6), lobular inflammation (0–4), and portal inflammation (0–4).

### Serum ALT measurements

ALT levels in serum were determined using a kit from Thermo Fisher Scientific following the manufacturer’s protocol. Briefly, serum samples were mixed with pre-warmed (37 °C) InfinityTM ALT (GPT) Liquid Stable Reagent at 1:10 ratio and OD readings were taken for 3 min at 1 min intervals in a nanodrop at a 340-nm wavelength, 37 °C. The slope was calculated by plotting absorbance vs. time using linear regression and multiplied with a factor to obtain ALT levels in serum (U/L) as described in the kit.

### Ex vivo cytokine secretion by iNKT cells, B cells, CD11b cells, and Kupffer cells

iNKT cells (TCRβint + αGalCer/CD1d tetramer + ) were sorted by flow cytometry from the livers, PCLNs and spleens of αGalCer/CD1d-NP- or Cys-NP-treated NOD.*c3c4* mice. The sorted cells were stimulated with anti-CD3/CD28 mAb-coated beads at 37 °C for 72 h. The levels of various cytokines and chemokines in the supernatants were measured via Luminex (for G-CSF, GM-CSF, Eoxtaxin, IFNγ, TNFα, IL-1α, IL-1β, IL-2, IL-3, IL-4, IL-5, IL-6, IL-7, IL-9, IL-10, IL-12p40, IL-12p70, IL-13, IL-15, IL-17, CXCL1, CXCL2, CXCL5, CXCL9, CXCL10, CCL2, CCL3, CCL4, and CCL5) and/or via ELISA (IL-10, IL-4, IL-21, and IFNγ) using commercially available kits (R&D Systems).

B cells from PCLN, MLN, spleen, liver and lung mononuclear cell suspensions from αGalCer/CD1d-NP- or Cys-NP-treated mice by cell sorting upon staining with PE-conjugated anti-CD19 mAb (see next section) or using an Easysep CD19 Positive Selection kit II (Stem Cell Technologies). The cells (2–3 × 10^5^ in 200 μL/well) were stimulated with LPS (1 μg ml^−1^, Sigma) for 24 h in RPMI-1640 media containing 10% FCS. The levels of the Breg cytokines IL-10 and IL-35 in the supernatants were measured via ELISA using IL-10 (R&D Systems) and IL-35 (Biomatik, Cambridge, ON) kits.

CD11b + cells were purified as previously described^22^. Briefly, LNs were digested in collagenase D (1.25 μg mL^−1^) and DNAse I (0.1 μg mL^−1^) for 15 min at 37 °C, washed, incubated with anti-FcR Abs, and the cell suspensions used to purify CD11b + cells using anti-CD11b mAb-coated magnetic beads (BD Biosciences). The purified cells (2–3 × 10^5^ in 200 μL/well) were stimulated with LPS (2 μg ml^−1^) for 3 days, and the supernatants analyzed for cytokine content using a Luminex multiplex cytokine assay.

To isolate Kupffer cells (KCs), liver single cell suspensions were subjected to a 37.5% Percoll gradient centrifugation in the presence of Heparin (10 U/ml), to separate RBCs and immune cells, including KCs, from non-immune liver cells. Upon hemolysis of RBCs using a red blood cell lysis solution (Miltenyi Biotec), KCs, were purified using F4/80+ microbeads (Miltenyi Biotec). The purified cells (2–3 × 10^5^ in 200 μL/well) were stimulated with LPS (2 μg ml^−1^) for 3 days, and the supernatants analyzed for cytokine content via Luminex.

### Mouse iNKT- and liver B cell purification

Mice were bled to completion by severing the heart and abdominal aortas. Liver cell suspensions were subjected to 37.5% isotonic Percoll gradient (Percoll, Sigma-Aldrich) centrifugation in the presence of heparin (10 U/ml) and mononuclear cells prepared as described above. Lungs were cut into small pieces and digested in RPMI-1640 medium containing 10% FCS, DNAseI (200 U/ml) and Collagenase IV (100 μg/ml) at 37^o^C for 90 mins. All the pieces were homogenized into a single cell suspension, washed and hemolyzed. Single cell suspensions from liver, lungs and spleen were stained with αGalCer/CD1d tetramer (3 to 5 μg/ml, at room temperature for 1 h) and anti-mouse TCRβ and B220 mAbs. For iNKTs, TCRβ int^+^B220-tetramer^+^ cells and for B cells, TCRβ^-^B220^+^ cells were sorted using a FACSAria III instrument (BD Biosciences). Dead cells were excluded from analysis by staining with 7ADD Viability dye from BD biosciences. The percent purity of the iNKT and B-cell preparations were: 88.3 ± 1.9 and 89.9 ± 0.76 for liver iNKTs of control vs. treated mice for bulk RNAseq; 94.6 ± 2.2 and 91.7 ± 0.96 for liver iNKTs of control vs. treated mice for scRNAseq; 98.1 ± 1 and 100 for liver B cells of control vs. treated mice for in vitro experiments. Unless indicated otherwise, mouse iNKT and B cells were isolated 2–3 days after the last αGalCer/CD1d-NP dose. In experiments that sought to measure the consumption rate of LiNTR1 cells, LiNKT cells were isolated 14 or 35 days after the last dose.

### Pancreatic islet preparation and isolation and culture and tetramer staining of islet-associated B cells or T cells

Pancreata from NOD mice treated with αGalCer/CD1d-NP or uncoated NP were injected with ~3 mL collagenase P (Millipore Sigma Cat# 11213857001, 0.66 mg/mL) through the pancreatic duct. The pancreata were then digested at 37 °C for 15 min and dispersed with pipetting. The islets were hand-picked under a stereomicroscope and incubated with IL-2-containig LCM for 2 h in a CO_2_ incubator. The islet cells and islet infiltrating mononuclear cells were further treated with trypsin at 37 °C for 3 min to make single cell suspensions. After Fc blocking, cells were stained with αGalCer/CD1d (for CyTOF) or InsB_13-21_/IA^g7^ tetramers at 37 °C or 4 °C, respectively, for 60 min in the presence of anti-CD4 and anti-B220 antibodies and a viability dye for the last 20 min.

### Human LiNKT cell purification

Intra-hepatic liver cell isolates (~20 million/sample) were stained with APC-labeled anti-human TCR-Vα24-Jα18 (1:20 dilution; Clone 6B11, Biolegend) in 100 μL of FACS buffer (1% FBS, 0.1% NaN_3_ in PBS) for 30 min at 4 °C. Stained cells were washed twice in 1 mL FACS buffer, resuspended in 80 μL of cold MACS buffer, incubated with 20 μL of anti-APC microbeads (Miltenyi Biotec) per 10^7^ total cells for 15 min at 4 °C, and magnetically purified on a MS column according to the manufacturer’s protocol. After positive magnetic isolation, the cells were washed and stained with 50μl αGalCer-hCD1d tetramer/antibody staining mix (FITC-labeled mouse anti-human CD3 (Clone HIT3a, Biolegend) at 1:10 dilution plus PE-labeled αGalCer-hCD1d tetramer (MBL) at 1:5 dilution) and incubated at 4 C in the dark for 20 min. 7-Aminoactinomycin D (7-AAD, BD Pharmingen) was used to discriminate between live and dead cells. After washing, the cells were resuspended in 500μl FACS buffer and FITC/PE-double-positive cells sorted by flow cytometry.

### In vitro suppression of antigen-induced T-cell proliferation by liver iNKTs

Liver KCs from untreated NOD.*c3c4* mice with established disease were purified using anti-F4/80 mAb-coated microbeads (Miltenyi Biotec) and pulsed with NRP-V7 peptide (2 μgml^−1^) for 2 h at 37 °C. After washing, peptide-loaded KCs (1.5 × 10^4^) were cultured for 6 days with purified splenic CD8 + T cells from 8.3-NOD.*G6pc2*^*–/–*^.*Tcra*^*–/–*^ mice (6 × 10^4^) (using BD IMag anti-mCD8 beads, BD Biosciences) at 1:4 ratio in the presence of liver iNKT cells (1.5 × 10^4^) sorted from αGalCer/CD1d-NP- or Cys-NP-treated NOD.*c3c4* mice, and mAbs (10μg/ml) against HRP (Rat IgG1, isotype control), IL-4 (11B11), IL-10 (JES5-2A5), TGFβ (1D11), IFNγ (BE0054), or IL-21Rα (4A9) (BioXcell, West Lebanon, NH). On day 2, BrdU was added to the cultures at a final concentration of 10 μM. On day 6, the CD8 + T cells in the cultures were examined by flow cytometry for BrdU-incorporation using the BD Pharmingen BrdU Flow kit.

### Ex vivo antigen-presenting capacity of liver KCs and PCLN CD11b + cells from treated mice

Liver KCs and portal and celiac LN (PCLNs) CD11b + cells from αGalCer/CD1d-NP- or Cys-NP-treated NOD.*c3c4* mice were purified using anti-F4/80 and anti-CD11b mAb-coated microbeads, respectively (Miltenyi Biotec, Auburn, CA) and pulsed with NRP-V7 peptide (2 μgml^−1^) for 2 h at 37 °C. After washing, peptide-loaded APCs (1.5 × 10^4^) were cultured for 6 days with purified splenic CD8 + T cells (6 × 10^4^) from 8.3-NOD.*G6pc2*^*–/–*^.*Tcr*α^*–/–*^ mice at a 1:4 ratio. On day 2, BrdU was added to the cultures at a final concentration of 10 μM. On day 6, the CD8 + T cells in the cultures were examined by flow cytometry for BrdU-incorporation using the BD Pharmingen BrdU Flow kit.

### CD1d-restricted agonistic activity of B cells on LiNKT cells

LiNKT cells from Cys-NP- and αGalCer/CD1d-NP-treated NOD.*c3c4* mice were co-cultured with NOD or NOD.*c3c4* mice-derived liver B cells at 1:1 ratio (4 × 10^4^ in 200 μL/well) at 37 °C, in the presence of isotype or anti-CD1d mAb (1 μg/mL). After 72 h, supernatants and cells were harvested. Absolute numbers and average MFI for CD69 on the LiNKTs were determined by flow cytometry. The levels of IL-10 and IL-21 in the supernatants were measured via ELISA as described above.

### Ex vivo antigen-presenting capacity of CD11b + cells from the pancreas-draining LNs of treated mice

APCs from PCLNs (draining both the pancreas and liver) of αGalCer/CD1d-NP- and Cys-NP-treated NOD mice were purified using anti-CD11b mAb-coated microbeads (Miltenyi Biotec). The purified cells were pulsed with NRP-V7 peptide (2 μgml^−1^) for 2 h at 37 °C. After washing, peptide-loaded DCs (1.5 × 10^4^) were cultured for 3 days with purified splenic CD8 + T cells (4.5 × 10^4^) from 8.3-NOD.*G6pc2*^*–/–*^.*Tcra*^*–/–*^ mice at 1:3 ratio. The supernatants were analyzed for IFNγ content via ELISA (R&D systems).

### In vivo Breg induction assay

Splenic B cells from NOD.*Il10*^*tm1Flv*^ (Tiger) mice were enriched using an EasySep Mouse B-cell Isolation Kit (Stem Cell Technologies), labeled with CellVue Claret Far Red Fluorescent Cell linker (Sigma, Oakville, Ontario, Canada) and transfused (3 × 10^6^) into αGalCer/CD1d-NP- or Cys-NP-treated mice. The hosts were killed 7 days later and their MLNs and PCLNs cells were labeled with anti-B220-BV420 and biotinylated anti-CD1d or anti-CD5 mAbs followed by Streptavidin-PerCP. CellVue Claret+ B cells were analyzed for presence of eGFP^+^CD1d^high^ and eGFP^+^CD5^+^ cells by flow cytometry.

### Suppression of antigen-presentation in vivo

Purified splenic 8.3-TCR-transgenic CD8 + T cells from 8.3-NOD.*G6pc2*^–/–^.*Tcra*^–/–^ donors (using BD IMag anti-mCD8 beads, BD Biosciences) were labeled with 2.5 μM CFSE (Molecular Probes, Eugene, OR), and adoptively transferred i.v. (5 × 10^6^ cells/mouse) into untreated NOD.*c3c4* mice, or NOD.*c3c4* mice that had been treated with 10 doses of αGalCer/CD1d-NPs or Cys-NPs i.v. twice weekly for 5 week. Six days later, spleen, PCLN, PLN, and MLN were collected, and the cell suspensions stained with anti-mCD8 mAb. Gated CD8 + T cells were analyzed for the extent of CFSE dilution using flow cytometry.

### Adoptive transfer of disease suppression

Liver iNKTs and liver and PCLN B cells from αGalCer/CD1d-NP- or Cys-NP-treated NOD.*c3c4* mice were sorted in flow cytometry or purified using a EasySep Mouse B-cell Isolation Kit, respectively. The purified iNKTs (0.2 × 10^6^ cells/mouse) or B cells (1 × 10^6^ cells/mouse) were adoptively transferred i.v. into 10–12-week-old NOD.*c3c4.scid* hosts. One day later, the recipients were adoptively transferred with 4 × 10^7^ pooled splenocytes and PCLN cells from sex-matched NOD.*c3c4* donor mice with established PBC (>24 week old). The recipients were euthanized 6 week later for tetramer staining and PBC scoring.

### In vivo cytokine and CD1d blockade

mAbs against HRP (Rat IgG1), IL-4 (11B11), IL-10 (JES5-2A5), TGFβ (1D11), or IFNγ (XMG1.2) were given i.p. twice a week at 500 μg/dose for 2 weeks, followed by 200 μg/dose for 3 additional weeks. To block iNKT-cell interactions with APCs, mAbs against HRP (Rat IgG1) or CD1d (CD1.1) were given i.p. twice a week at 500 μg/dose for 2 weeks, followed by 200 μg/dose for 3 additional weeks. All the mAbs were from BioXcell (West Lebanon, NH).

### Effects on normal immunity

Cellular immunity to intracellular bacteria was determined by infecting αGalCer/CD1d-NP- or Cys-NP-treated NOD.*c3c4* mice i.v. with 10^3^ colony forming units (cfu) of *Listeria monocytogenes* (LM) (DMX Corporation, Philadelphia, PA). The mice were euthanized 3 or 35 days later to determine bacterial load in liver and spleen. Briefly, spleen and liver were cut into several pieces, weighted and homogenized in PBS containing 0.35% Triton X-100. Serial dilutions of the lysates were then plated onto Bovine Heart Infusion agar containing 5 μg/ml erythromycin, incubated for 24-48 h at 37 °C and the number of colonies counted.

To evaluate humoral immunity, αGalCer/CD1d-NP- or Cys-NP-treated NOD.*c3c4* mice were immunized i.p. with 100 μg of DNP-KLH (Alpha Diagnostic International, San Antonio, TX) in CFA and boosted again 3 week later as previously described^23^. Mice were euthanized 10 days later, to measure serum anti-DNP antibody titers using an anti-DNP Ig ELISA Kit (Alpha Diagnostic International).

### Bulk RNAseq

Cells were incubated with lysis buffer (10^5^ cells) to perform RNA extractions for RNAseq. Total RNA was prepared using a RNeasy Plus Mini Kit (Qiagen, Hilden, Germany) and used for preparation of RNAseq libraries and sequencing (Centre for Genomic Regulation, Barcelona, Spain). Libraries were prepared using the TruSeq Stranded mRNA Sample Prep Kit v2 according to the manufacturer’s protocol (Illumina, San Diego, CA). Briefly, 10–50 ng of total RNA was used for poly(A)-mRNA purification using streptavidin-coated magnetic beads followed by fragmentation to ~300 bp. cDNA was synthesized using reverse transcriptase (SuperScript II, Invitrogen) and random primers. The second strand of the cDNA incorporated dUTP in place of dTTP. Double-stranded DNA was further used for library preparation. dsDNA was subjected to A-tailing and ligation of the barcoded Truseq adapters. All purification steps were performed using AMPure XP Beads (Beckman Coulter, Brea, CA, USA). Library amplification was performed by PCR using the primer cocktail supplied in the kit. Final libraries were analyzed using Agilent DNA 1000 chip to estimate the quantity and size distribution and were then quantified by qPCR using the KAPA Library Quantification Kit (Kapa Biosystems, Wilmington, MA) prior to amplification with Illumina’s cBot. Libraries were loaded at a concentration of 2.75 pM onto the flowcell, and were sequenced 1 ×50 on Illumina’s HiSeq 2500 to obtain 30–40 M reads.

### Single cell RNAseq

Purified liver iNKT cells from αGalCer/CD1d-NP- or untreated NOD.*c3c4* mice were first pooled and then partitioned into Gel Bead-In-Emulsions with a Target Cell Recovery of ~4000 total cells. Cell number and viability were verified using an automated cell counter (Moxi Flow Next Gen Flow Cytometer, ORFLO Technologies). Four single cell 3′ gene expression libraries (*n* = 2 libraries from control treated mice, *n* = 2 libraries from αGalCer/CD1d-NP-treated mice) were prepared using the Chromium Next GEM single cell 3′ reagent v3.1 and gel bead kits from 10x Genomics. hLiNKTs isolated from 3 human liver explants were processed simultaneously as described above for the mouse samples (in one case along with sorted tetramer-negative CD3 + cells, later excluded form analysis) and sequenced together. Appropriate volume of cells, as determined from the user guide for recovery of ~4000 cells, was loaded on the Chromium single cell controller chip. cDNA QC and quantification and library construction QC were done using an Agilent Bioanalyzer high sensitivity DNA chip for use with the Agilent 2100 Bioanalyzer. Prepared libraries were quantified using the Qubit dsDNA HS Assay kit on the Qubit fluorometer. For sequencing, all 4 libraries were pooled and loaded at 300 pM on the NovaSeq Ilumina system using a NovaSeq SP flowcell. A 28 base-pair Read 1 was used to sequence the cell barcode and UMI, an 8 bp i7 index read was used to sequence the sample indexes and a 91 bp Read 2 was used to sequence the transcripts using paired-end sequencing.

### Bioinformatics

Bioinformatic analyses of the bulk RNAseq data were done using Partek Flow software (Partek Inc., St. Louis, MO). For RNAseq, the quality of the fastq files was determined using the FastQC software (FastQC). Contaminant rRNA was filtered using Bowtie 2 (2.2.5). Reads were aligned with the STAR mapper (version 2.5.3a) to the GENCODE release 16 of the *Mus musculus genome* (mm10 assembly)^74^. A raw count of reads per gene was obtained with Partek “Quantify to annotation model (Partek E/M)” tool. DESeq2^75^ was used to assess differential expression between experimental groups (Wald statistical test + False Discovery Rate correction). A threshold of a Fold-Change ≥2 or ≥4 and a FDR ≤ 0.01 were used to filter the genes that were differentially expressed between populations. Further analyses were performed in R Studio. Packages used include UpSetR and pheatmap.

For 10x scRNAseq, Single cell transcriptome sequencing data was processed using the Cell Ranger version 3.1 software. Briefly, the fastq files were processed with the ‘cellranger count’ pipeline, which uses STAR mapper, with either the mouse mm10 or the human GRCh38 human reference transcriptomes. The ‘cellranger aggr’ pipeline was run to generate an expression matrix with the combined libraries with normalization setting set to ‘None’. The expression matrix generated was then analyzed using the package ‘Seurat’ v3 in R^76^. Data was filtered based on QC metrics by genes present in >3 cells, cells with features between 200 and 5000 and percent of mitochondrial genes <20%. The data was then log normalized and scaled. A PCA-reduction was performed and 30 significant PCA-dimensions were taken into account. Clusters were determined using the kmeans function setting the number of centers to 3. Cluster annotation was done manually based on the expression of lineage-specific hallmark genes. Differentially expressed genes for one cluster (versus all cells in other clusters) was determined by a negative binomial generalized linear model.

### Cytoscape

Graphical representation was performed using Cytoscape version 3.2.1^77^. Genes were represented as circular/rectangular nodes and arrows used to represent the direction of the regulatory function. In circular plots, a degree sorted layout was used to represent the nodes with highest degree of connectivity at the bottom of the circle and decreasing as proceeding counter-clockwise around the circle. See the figure captions for additional details.

### Statistical analyses

Unless specified, sample size values mentioned in the figure legends correspond to the total number of mice examined, pooled from different experiments. Data were compared in GraphPad Prism v6-v9 using Mann-Whitney *U*-test, two-way ANOVA or one-way ANOVA or Multiple *t*-tests using the Tukey’s or Dunnet’s and Holm-Sidak’s corrections for multiple comparisons, respectively. *P* values < 0.05 were considered statistically significant. Only statistically significant *P* values are shown on Figures.

### Reporting summary

Further information on research design is available in the Nature Research Reporting Summary linked to this article.

## Supplementary information

Supplementary Information

Description of additional Supplementary File

Supplementary Data 1

Supplementary Data 2

Supplementary Data 3

Supplementary Data 4

Supplementary Data 5

Supplementary Data 6

Reporting Summary

## Data Availability

Source data are provided as a Source Data file. The following datasheets can be found online: Supplementary Data 1: Transcriptional relationships of LiNKT cells with iNKT cell subsets; Supplementary Data 2: Differential gene expression between LiNKT cells from NOD.c3c4 mice and B6 or NOD mice; Supplementary Data 3: Normalized gene expression counts in LiNKT cells from NOD.c3c4, B6, and NOD mice; Supplementary Data 4: Normalized gene expression counts in LiNKT cells from αGalCer/CD1d-NP-treated vs. control NOD.*c3c4* mice; Supplementary Data 5: Normalized gene expression counts in LiNKT cells from αGalCer/CD1d-NP-treated vs. control NOD.*c3c4* mice, for the genes listed in Tables 1 and 2; Supplementary Data 6: Transcriptional relationship between αGalCer/CD1d-NP-induced LiNKTR1 cells and pMHCII-NP-induced TR1 CD4 + T cells. The raw RNAseq and scRNAseq data files have been uploaded to the GEO database (accession number: GSE168488). Bulk RNA reads were aligned to the GENCODE release 16 of the *Mus musculus genome* (mm10 assembly). scRNAseq fastq files were processed using either the mouse mm10 or the human GRCh38 human reference transcriptomes.

## Source data

Source Data
