# Supplementary material for: Re-programming mouse liver-resident invariant natural killer T cells for suppressing hepatic and diabetogenic autoimmunity
Source: Nat Commun. 2022 Jun 7;13:3279. doi: 10.1038/s41467-022-30759-w (PMC9174212; doi:10.1038/s41467-022-30759-w)
Supplement: Supplementary file 1 — Supplementary Information [file 41467_2022_30759_MOESM1_ESM.pdf]

SUPPLEMENTARY INFORMATION

RE-PROGRAMMING MOUSE LIVER-RESIDENT INVARIANT NATURAL KILLER T CELLS FOR SUPPRESSING HEPATIC AND DIABETOGENIC AUTOIMMUNITY

Channakeshava Sokke Umeshappa et al.

Correspondence: P. Santamaria (psantama@ucalgary.ca)

Supplementary Figure 1

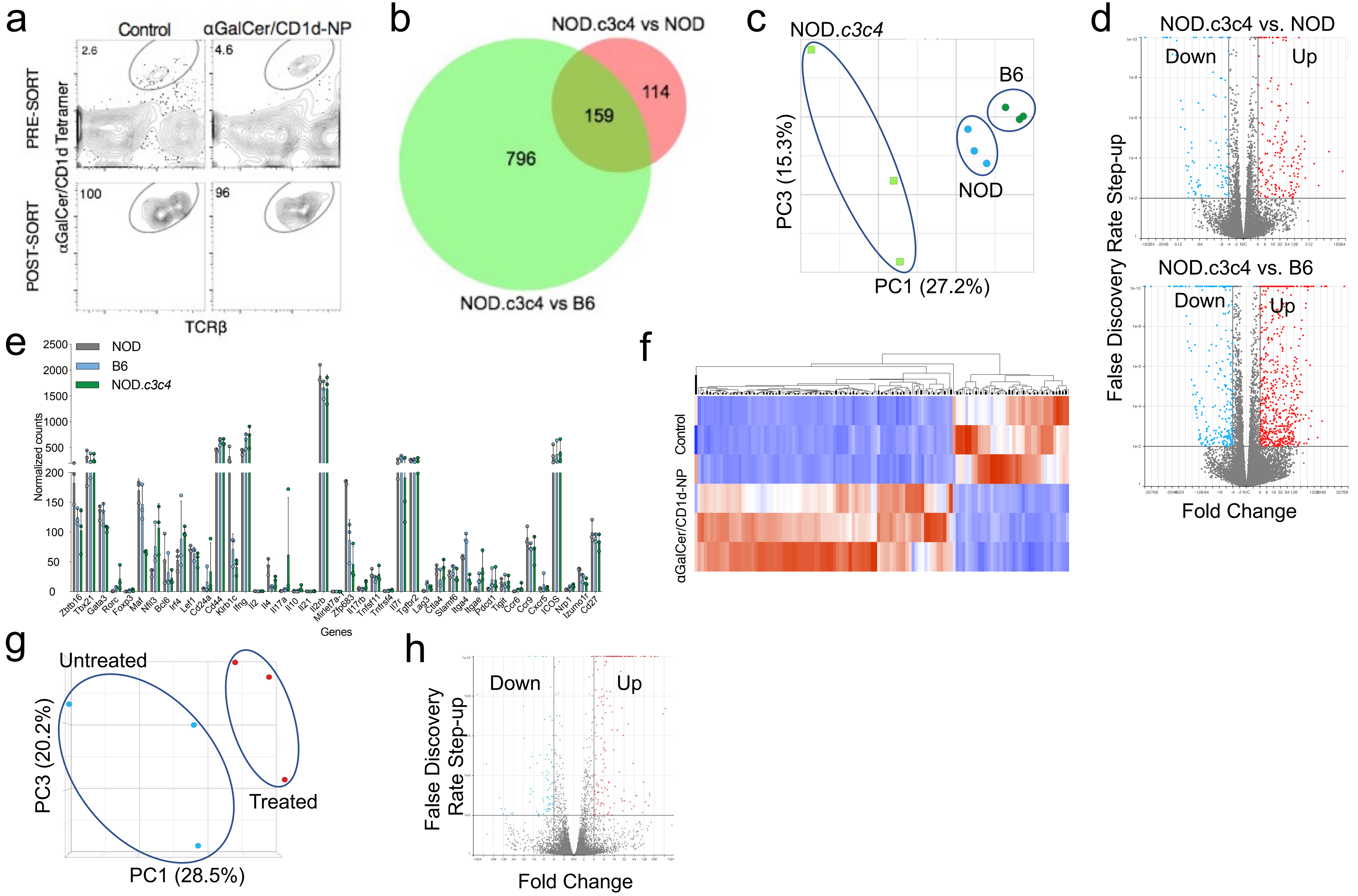

Supplementary Figure 1. **Transcriptional profiling of liver-resident iNKT cells in control vs. αGalCer/CD1d-NP-treated mice. a.** Representative flow cytometric profiles of LiNKTs sorted from control (untreated) and αGalCer/CD1d-NP-treated NOD.*c3c4* mice. Top and bottom panels show pre- and post-sort αGalCer/CD1d tetramer vs TCRβ staining profiles. **b**, Venn diagram comparing differentially expressed genes in LiNKT cells from untreated NOD.*c3c4* vs. B6 or NOD mice. **c**, Principal component analysis (PCA) plot showing LiNKT of untreated NOD (blue), B6 (dark green) and NOD.*c3c4* (light green) mice. **d**, Volcano plots from genes in the comparison of LiNKT of untreated NOD.*c3c4* vs. NOD mice (top) and untreated NOD.*c3c4* vs. B6 mice (bottom). X axis shows FC and vertical axis shows False discovery rate (FDR). Threshold for significance is set at FC$\geq$4 and FDR$\leq$0.01. In red, genes significantly upregulated in NOD.*c3c4* vs. B6 and NOD mice (n=657 in NOD.*c3c4* vs. B6; n=174 in NOD.*c3c4* vs. NOD); in blue, downregulated genes (n=298 in NOD.*c3c4* vs. B6; n=99 in NOD.*c3c4* vs. NOD). **e**, Histogram of normalized counts (normalization by counts per million (CPM)) of the LiNKT cell subset gene selection from Suppl. Table 1 comparing LiNKT of different untreated mice strains. **f**, Heatmap showing differentially expressed genes (FC$\geq$4 and FDR$\leq$0.01) in the comparison of LiNKT of control vs. αGalCer/CD1d-NP-treated mice. In red, upregulated genes for each population; in blue, downregulated genes. **g**, PCA plot showing LiNKT of treated (red) and untreated (blue) mice. **h,** Volcano plot from genes in the comparison of LiNKT of treated and untreated mice. X axis shows FC and vertical axis shows FDR. Threshold for significance is set at FC$\geq$4 and FDR$\leq$0.01. In red, genes significantly upregulated in treated vs. untreated (n=162); in blue, downregulated genes (n=76). Data are represented as means ± SEM and were compared using one tail Mann-Whitney U test (**a**) or multiple t-test comparisons corrected using the Holm-Sidak method (**b**). *P < 0.05; **P < 0.01; ***P < 0.001; ****P < 0.0001.

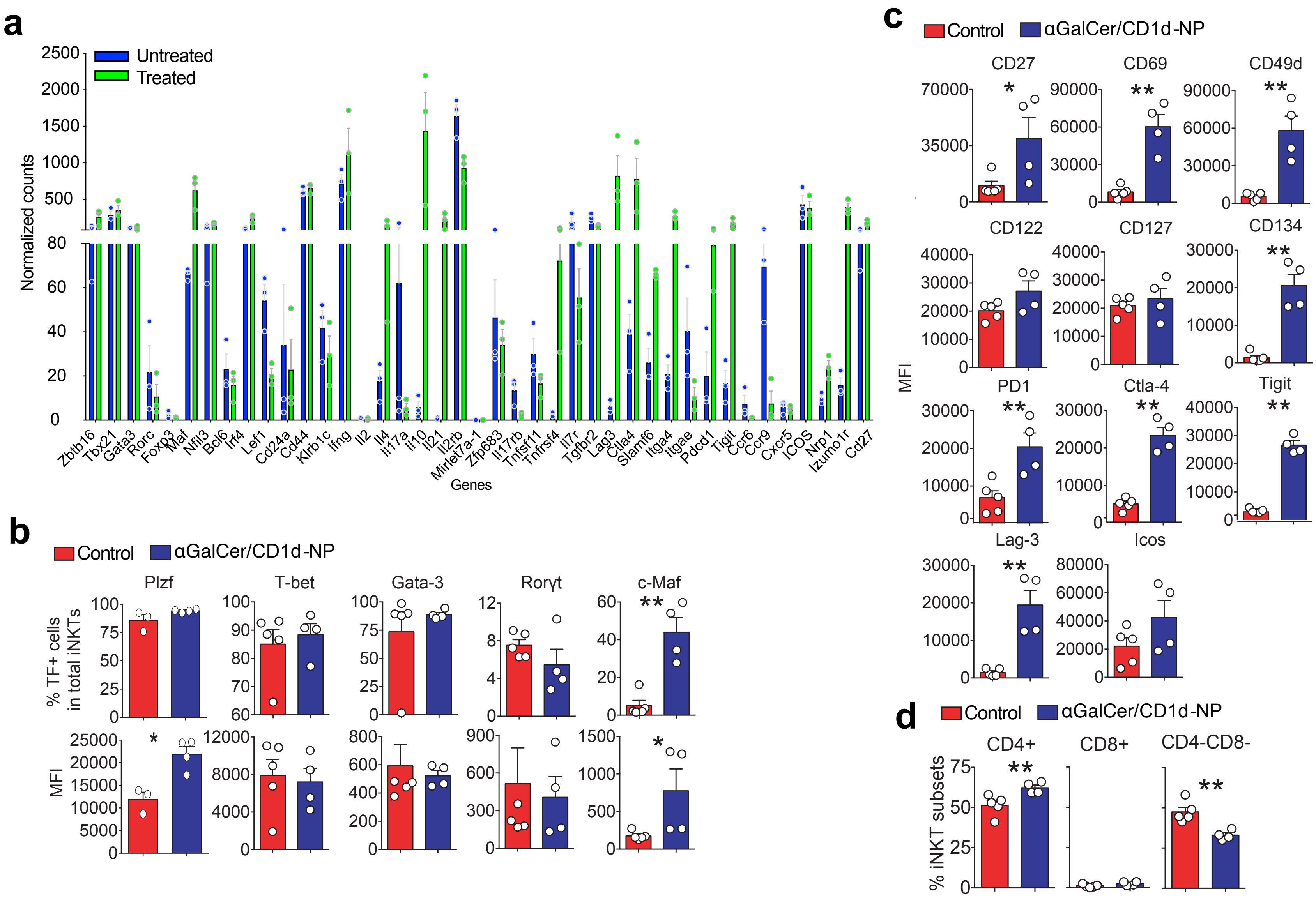
a
Untreated
Treated
Normalized counts
Genes
Zbtb16
Tbx21
Gata3
Rorc
Foxp3
Maf
Nfil3
Bcl6
Irf4
Lef1
Cd24a
Cd44
Klrb1c
Ifng
Il2
Il4
Il17a
Il10
Il21
Il2rb
Mirlet7a-1
Zfp683
Il17rb
Tnfsf11
Tnfrsf4
Il7r
Tgfbr2
Lag3
Ctla4
Slamf6
Itga4
Itgae
Pdcd1
Tigit
Ccr6
Ccr9
Cxcr5
ICOS
Nrp1
Izumo1r
Cd27
b
Control
αGalCer/CD1d-NP
Plzf
T-bet
Gata-3
Rorγt
c-Maf
% TF+ cells in total iNKTs
MFI
c
CD27
CD69
CD49d
CD122
CD127
CD134
PD1
Ctla-4
Tigit
Lag-3
Icos
d
CD4+
CD8+
CD4-CD8-
% iNKT subsets

Supplementary Figure 2. **Transcriptional and phenotypic re-programming of liver-resident iNKT cells in response to αGalCer/CD1d-NP therapy. a**, Histogram of normalized counts (CPM) in LiNKT of treated vs. untreated mice for the genes from Suppl. Table 1. **b,** Average percentages and MFI for TF staining in LiNKTs of control (n=5, untreated) and αGalCer/CD1d-NP-treated NOD.*c3c4* mice (n=4). **c**, Average MFI staining for iNKT cell surface markers of control (n=5, untreated) and αGalCer/CD1d-NP-treated NOD.*c3c4* mice (n=4). **d**, Effects of αGalCer/CD1d-NP treatment on the relative frequencies of CD4+CD8–, CD4–CD8+ and CD4–CD8– LiNKT cell subsets. Data correspond to 5 control (untreated) and 4 αGalCer/CD1d-NP-treated NOD.*c3c4* mice. Data are represented as means ± SEM and were compared using one tail Mann-Whitney U test (**b-d**) or multiple t-test comparisons corrected using the Holm-Sidak method (**a**). *P < 0.05; **P < 0.01; ***P < 0.001; ****P < 0.0001.

Supplementary Figure 3

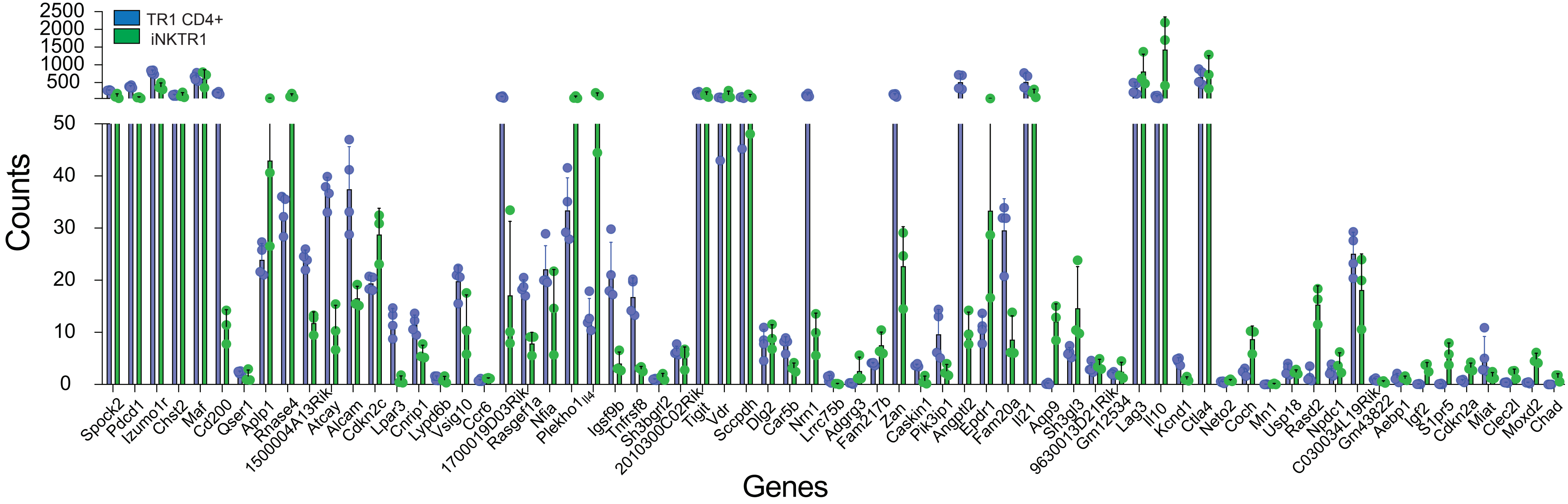

Supplementary Figure 3. LiNKTR1 vs. CD4+ TR1 transcriptomes. The LiNKT cells from αGalCer/CD1d-NP-treated vs. untreated NOD.*c3c4* mice and the tetramer+ (TR1) vs. tetramer– CD4+ T-cells (T-conventional) from BDC2.5mi/IA^{g7}-NP-treated NOD mice shared 66 differentially-expressed genes ($|FC|\geq4$, $FDR\leq0.01$). This figure compares the normalized counts (CPM) for these 66 genes (average ± SEM). In blue, counts from TR1 cells; in green, counts from LiNKTR1 cells. Data were compared via multiple t-test comparisons corrected using the Holm-Sidak method.

Supplementary Fig. 4

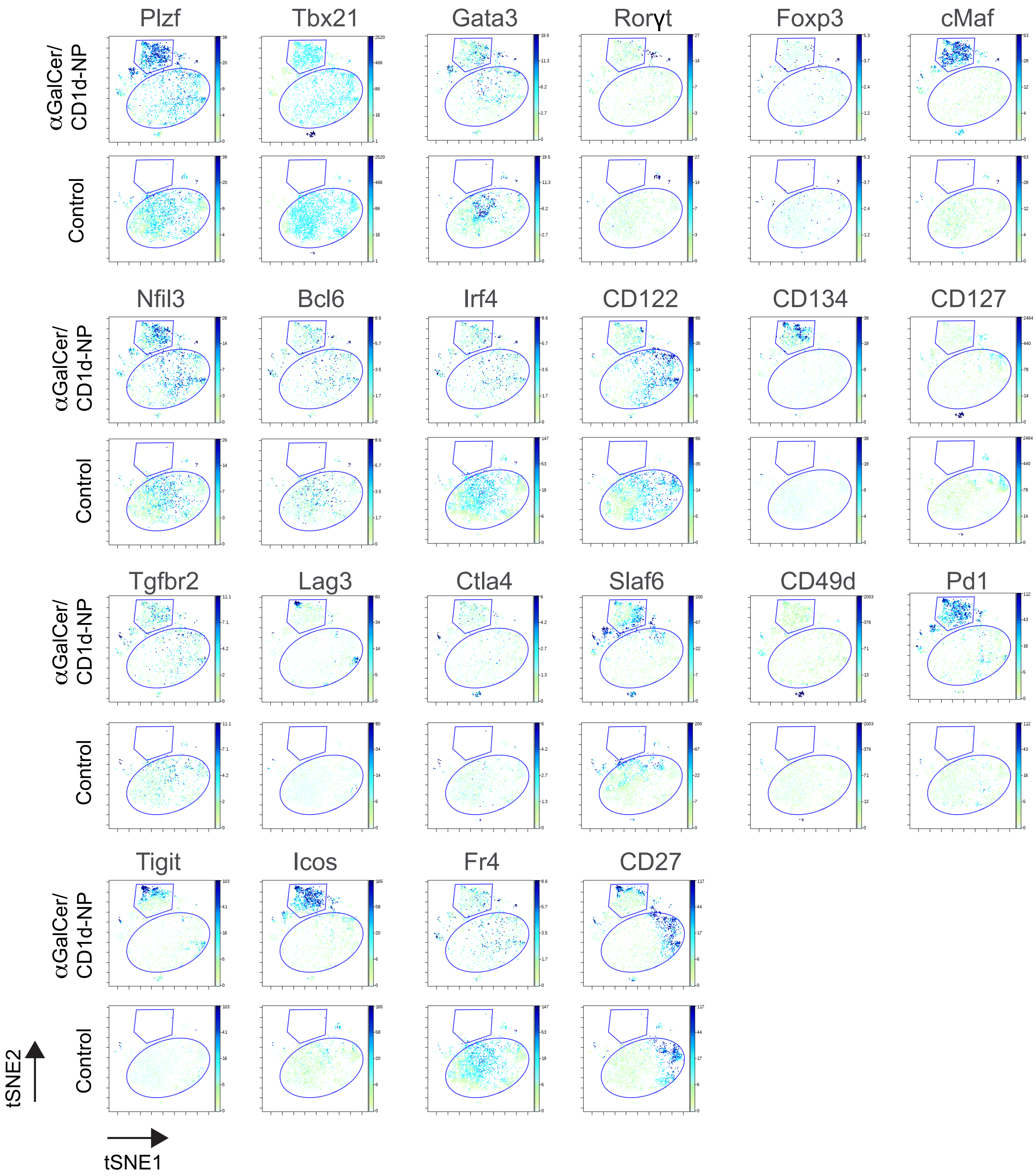

Supplementary Figure 4. **Mass cytometry plots for 22 LiNKT-relevant markers**. Data correspond to Main Fig. 4E.

Supplementary Figure 5

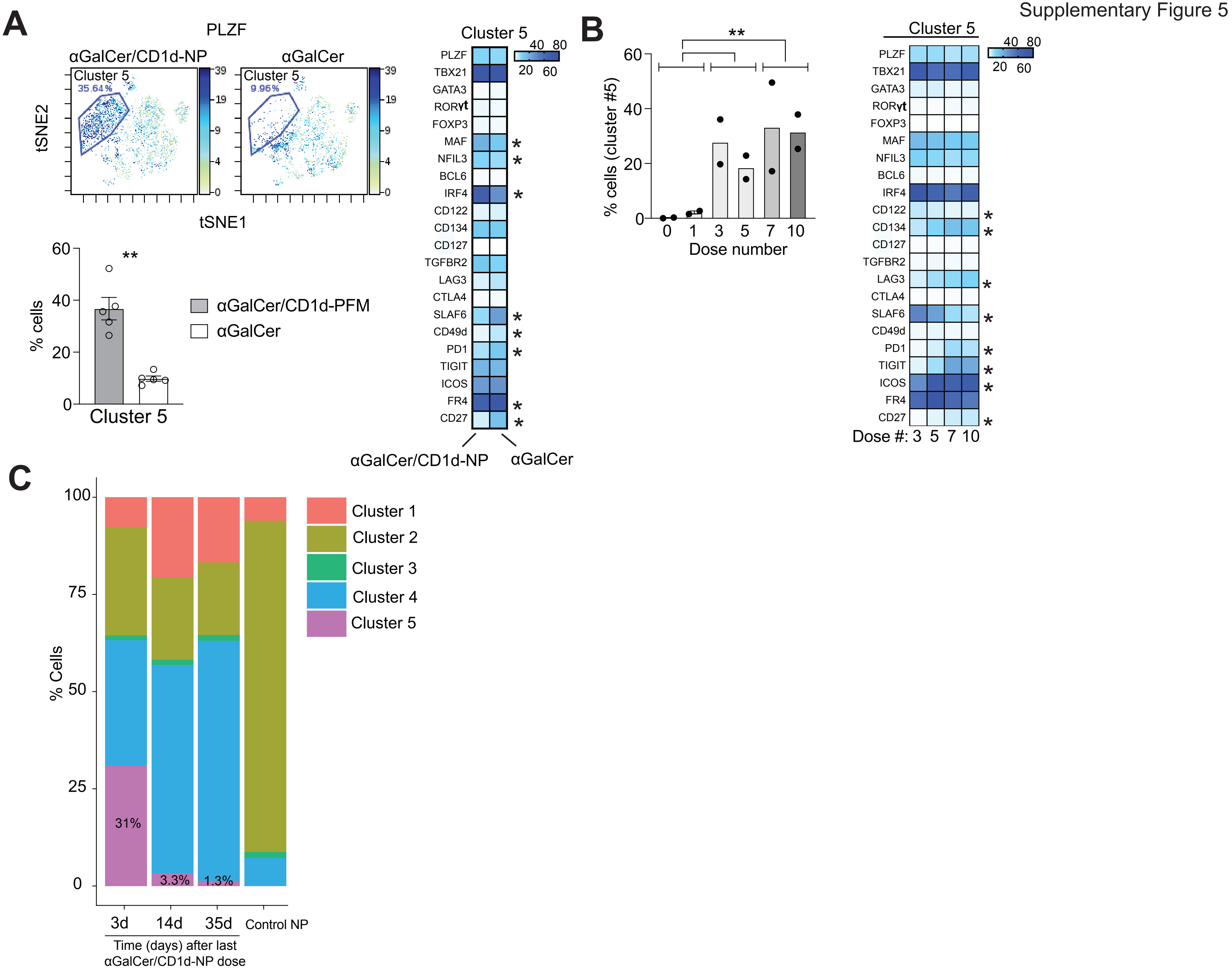

Supplementary Figure 5. Ability of αGalCer/CD1d-NP vs. αGalCer lipid to trigger LiNKTR1 cell formation, dose requirements and LiNKTR1 persistence. a, Cluster analyses of αGalCer/CD1d tetramer+ cells from the livers of NOD mice treated with αGalCer or αGalCer/CD1d-NPs (n=5 each), upon staining with 22 metal-labeled antibodies and analysis via mass cytometry. Top panel shows representative tSNE plots (for PLZF), indicating the position of the cluster 5 identified via scRNAseq. Bottom panel, average percentage of cluster #5 (LiNKTR1) cells in each treatment group. Right panel corresponds to expression heatmaps for each marker in the LiNKTR1 cells arising in each treatment group. Asterisks indicate markers with statistically significant differences in labelling intensity. **b**, Average percentages of cluster #5 (LiNKTR1) cells arising in mice treated with 0, 1, 3, 5, 7 or 10 doses of αGalCer/CD1d-NPs (n=2 pools of 2 mice each/dose number). Right panel corresponds to expression heatmaps for each marker in the LiNKTR1 cells arising in each treatment group. Asterisks indicate markers with statistically significant differences in labelling intensity. **c,** Changes in the percentage of clusters #1-5 with time after the last dose, as measured via scRNAseq. Data in a (bottom) corresponds to the average ± SEM. P values in **a** and **b** were calculated via one tail Mann-Whitney U. In **b**, we compared differences between values for times 0 and 1, 3 and 5 and 7 and 10, respectively. Data in **a-c** were generated using cells isolated 2-3 days after the last αGalCer/CD1d-NP dose, except in **c**, where samples were also collected 14 or 35 days after the last dose.

SUPPLEMENTARY FIGURE 6

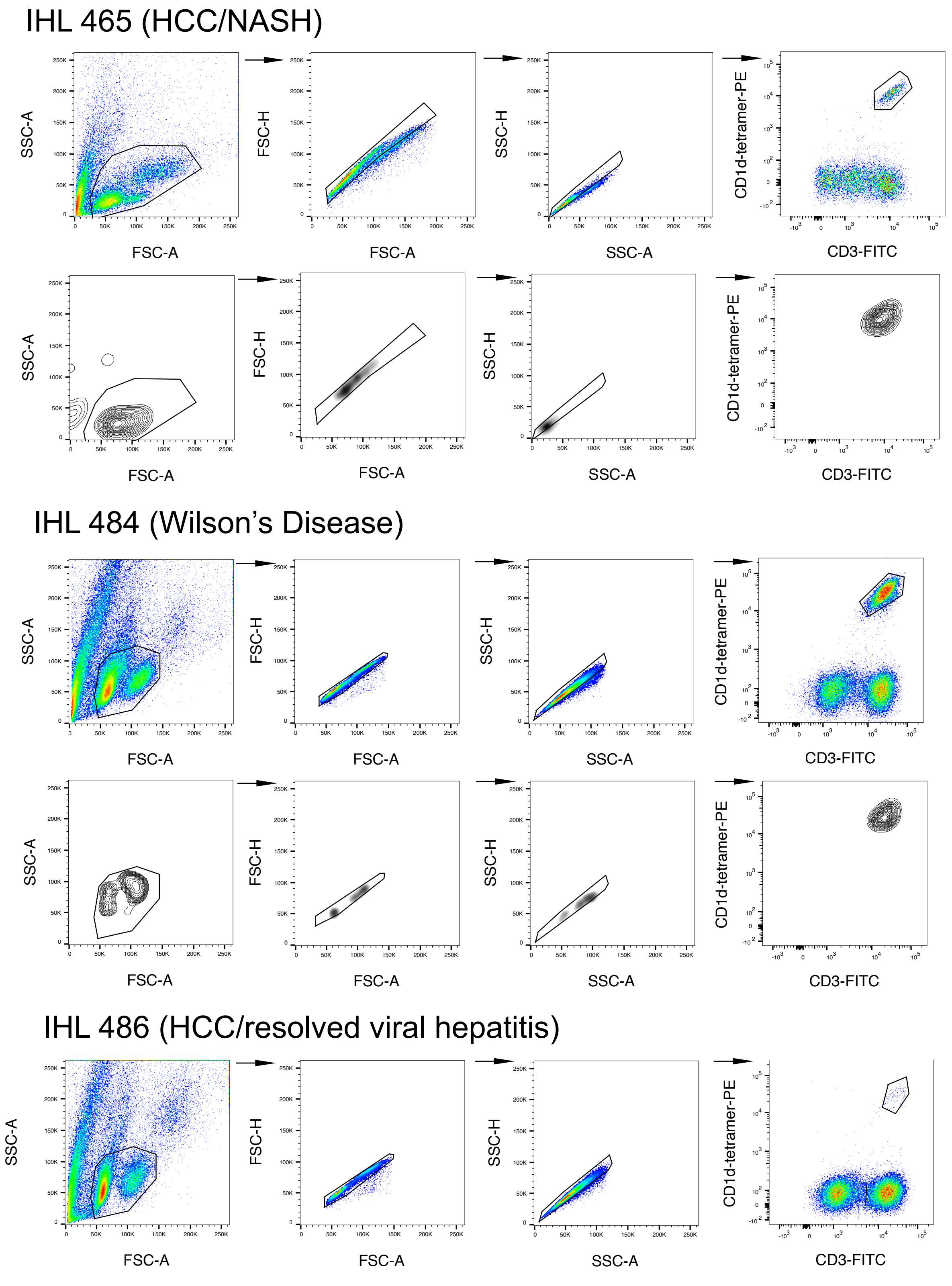

Supplementary Figure 6. **Pre- and post-sorting flow-cytometric profiles of LiNKT-cell-enriched isolates from human liver explants**. The post-sort profile for IHL-486 was not recorded. The number of total viable cells recovered from each patient, the total number of cells recovered after anti-Vα24Jα18 enrichment, and the total number of T-cells that were αGalCer/CD1d tetramer+ were as follows. IHL-565: 8.4×10^6, 2×10^5 and 1,092, respectively; IHL-484: 1.09×10^7, 1.5×10^5 and 159, respectively; IHL-486: 2.25×10^7, 0.5×10^5 and 152, respectively.

SUPPLEMENTARY FIGURE 7

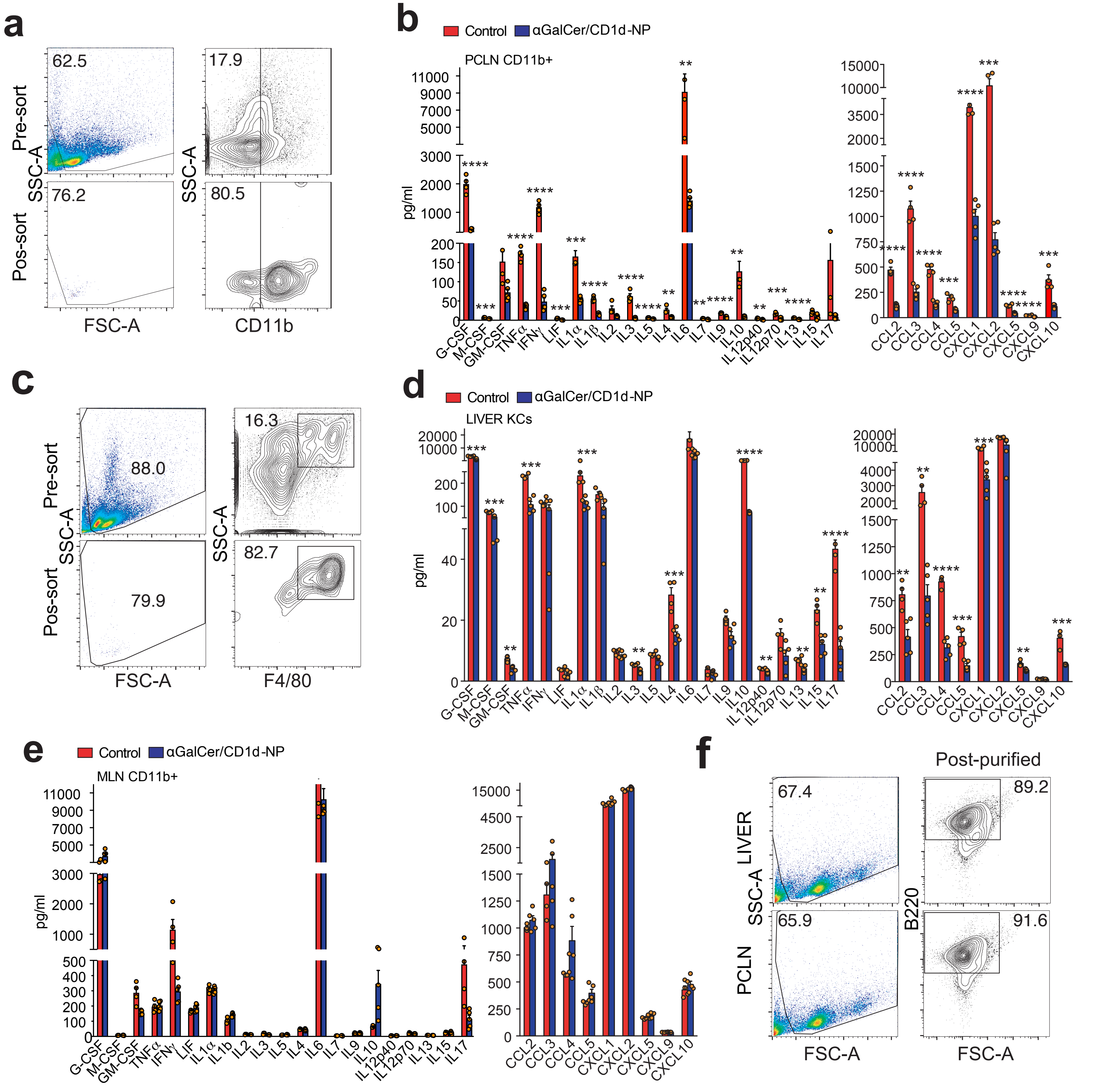

Supplementary Figure 7. Flow cytometry gating schemes for CD11b, KC and B-cell isolation and cytokine profiles for CD11b and KCs. a, c and f, Representative flow profiles for CD11b+ cells (**a**), KCs (**c**) and B-cells (**f**) before and/or after sorting. **b-e**, Cytokine/chemokine secretion by CD11b+ cells purified from the PCLNs (**b**) or MLNs (**e**) and liver KCs (**d**) of control (n=4, Cys-NP-treated) and αGalCer/CD1d-NP-treated NOD.*c3c4* mice (n=5). Data in **b-e** correspond to means ± SEM. P values were calculated using multiple one tail t-test comparisons corrected using Holm-Sidak method.

SUPPLEMENTARY FIGURE 8

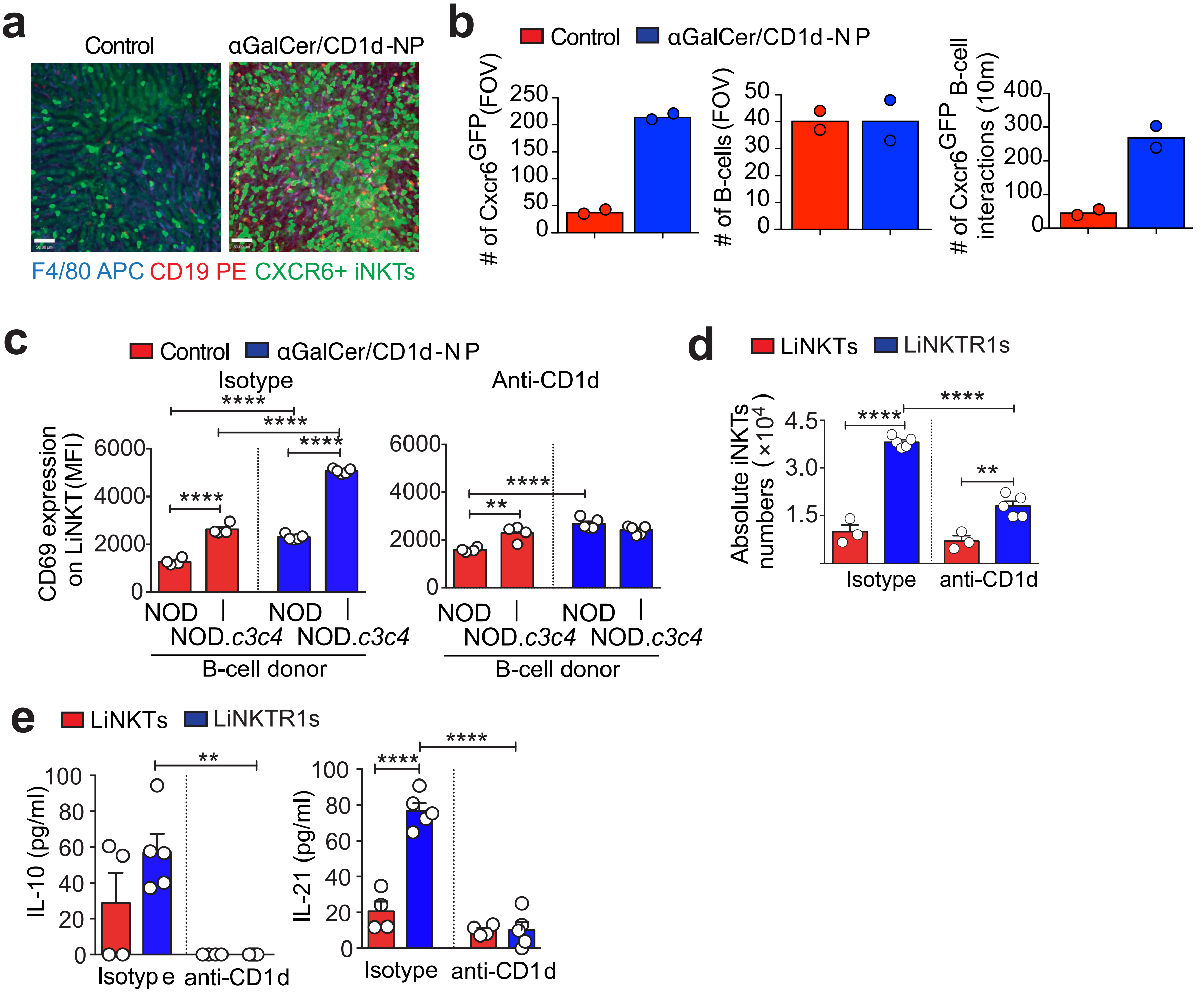

Supplementary Figure 8. Cognate interactions between LiNKTR1 cells and B-cells. a, Representative images showing increased number of LiNKTs (CXCR6-eGFP+) and B-cells as well as their interactions in the livers of Cxcr6$^{GFP/+}$ B6.*Ifng-ARE-Del*$^{+/–}$ mice in response to αGalCer/ CD1d-NP treatment. Light green autofluorescence: hepatocytes; Dark green (eGFP+): CXCR6+ iNKTs; Blue (anti-F4/80): Kupffer cells; Red (anti-CD19): B-cells. Scale bars, 50 µm. **b,** Average number of accumulated LiNKTs (CXCR6 eGFP+) and B-cells in the field of view (FOV) and their interactions in the liver in response to αGalCer/CD1d-NP- vs. Cys-NP treatment (n=2/group). **c,** Average MFI for CD69 on the LiNKTs of Cys-NP- (n=4) and αGalCer/CD1d-NP-treated (n=5) NOD.*c3c4* mice upon stimulation with liver B cells from NOD or NOD.*c3c4* mice, in the presence of isotype or anti-CD1d mAb. **d,** Average number of LiNKTs from Cys-NP (n=3) vs. αGalCer/CD1d-NP-treated (n=5) mice upon stimulation with liver B-cells from NOD.*c3c4* mice in the presence of isotype or anti-CD1d mAb. **e,** Average levels of IL-10 and IL-21 secreted by the LiNKTs of Cys-NP (n=4) and αGalCer/CD1d-NP-treated (n=5) mice in response to co-culture with liver B-cells from NOD.*c3c4* mice in the presence of isotype or anti-CD1d mAb. Data correspond to mean ± SEM. P values were calculated using one-way ANOVA with Tukey's post hoc correction (**c-e**). *P < 0.05; **P < 0.01; ***P < 0.001; ****P < 0.0001.

Supplementary Table 1. List of reagents, mice and the corresponding catalog numbers

REAGENT	SOURCE	Catalog#	Conc. (μg/ml) or dilution
**Antibodies and Tetramers**			
Anti-CD5 antibody, Biotin Conjugated, Clone 53-7.3	BD Biosciences	Cat# 553019	5
Anti-CD19 antibody, Phycoerythrin Conjugated, Clone 1D3	BD Biosciences	Cat# 553786	2
Anti-CD27 antibody, Phycoerythrin Conjugated, Clone LG.3A10	BD Biosciences	Cat# 553235	2
Anti-CD69 antibody, Biotin Conjugated, Clone H1.2F3	BD Biosciences	Cat# 553235	2
Anti-CD49d antibody, Phycoerythrin Conjugated, Clone 9C10 (MRF4.B)	BD Biosciences	Cat# 557420	2
Anti-CD122 antibody, Phycoerythrin Conjugated, Clone TM-beta 1	BD Biosciences	Cat# 553362	2
Anti-CD134 antibody, Biotin Conjugated, Clone OX-86	BD Biosciences	Cat# 559862	5
Anti-CD152 (CTLA-4) antibody, PE Conjugated, Clone UC10-4F10-11	BD Biosciences	Cat# 553720	2
Anti-CD1d antibody, Biotin Conjugated, Clone 1B1	BD Biosciences	Cat# 553844	5
Anti-CD11b antibody, FITC Conjugated, Clone M1/70	BD Biosciences	Cat# 557396	5
Anti-Mouse CD45R/B220 antibody, BV421 conjugated, Clone RA3-6B2	BD Biosciences	Cat#; 562922	2
Anti-CD45R/B220 antibody, PerCP Conjugated, Clone RA3-6B2	BD Biosciences	Cat# 553093	2
Anti-CD45R/B220 antibody, Allophycocyanin Conjugated, Clone RA3-6B2	BD Biosciences	Cat# 553092	2
anti-mouse CD223 (LAG-3) antibody, PE conjugated, Clone C9B7W	BD Biosciences	Cat# 552380	2
anti-ICOS (CD278) antibody, BV421 conjugated, Clone C398.4A	BD Biosciences	Cat# 565887	2
Anti-TCRbeta antibody, FITC Conjugated, Clone H57-597	BD Biosciences	Cat# 553171	5
Anti-PLZF antibody, PE conjugated, Clone R17-809	BD Biosciences	Cat# 564850	2
Anti-human GATA3 Antibody, PE-Cy7 Conjugated, clone L50-823	BD Biosciences	Cat# 560405	1:5 dilution
Anti-Mouse RORγt antibody, PE conjugated, Clone Q31-378	BD Biosciences	Cat# 562607	2
Anti- mouse CD16/CD32 antibody, Unconjugated, Clone 2.4G2	BD Biosciences	Cat# 553141	5
7-AAD Staining Solution	BD Biosciences	Cat# 559925	1:1000 dilution
Anti-mouse TIGIT antibody, PE conjugated, Clone GIGD7	Invitrogen	Cat# 12-9501-82	2
Anti-mouse c-MAF antibody, PE conjugated, Clone sym0F1	Invitrogen	Cat# 12-9855-42	2

Anti-mouse CD4 antibody, PE/Cyanine7 conjugated<br>Clone RM4-5	Biolegend	Cat# 100527	2
Anti-mouse CD8a antibody, PE conjugated, Clone 53-6.7	Biolegend	Cat# 100707	2
Anti-mouse CD279 (PD-1) antibody, PE conjugated, Clone 29F.1A12	Biolegend	Cat# 135205	2
Anti-T-bet antibody, Brilliant Violet 421 conjugated, Clone 4B10	Biolegend	Cat# 644815	2
F4/80 antibody, FITC conjugated, Clone BM8	eBioscience™	Cat# 11-4801-82	5
F4/80 antibody, APC conjugated, Clone BM8	eBioscience™	Cat# 47-4801-82	2
Anti-CD19 antibody, PE conjugated, clone 1D3	Pharmingen	Cat# 553786	2
Anti-mouse CD127 antibody, PE conjugated, Clone A7R34	eBioscience™	Cat# 12-1271-82	2
InVivoMab rat IgG1 isotype control (anti-HRP) antibody, Purified, Clone HRPN	Bio X Cell	Cat# BE0088	10
InVivoMab anti-mouse IL-4 antibody, Clone 11B11	Bio X Cell	Cat# BE0045	10
InVivoMab anti-mouse IL-10 antibody, Purified, Clone JES5-2A5	Bio X Cell	Cat# BE0049;	10
InVivoMab anti-mouse IL-21R antibody, Purified, Clone 4A9	Bio X Cell	Cat# BE0258;	10
InVivoMab anti-mouse/human/rat/monkey/hamster/canine/bovine TGFβ antibody, Purified, Clone 1D11.16.8	Bio X Cell	Cat# BE0057;	10
InVivoMab anti-mouse IFN-γ antibody, Clone XMG1.2	Bio X Cell	Cat# BE0045	10
Anti-human TCR-Vα24-Jα18 antibody, APC conjugated, clone 6B11	Biolegend	Cat# 342907	2
Anti-cMAF antibody (mass cytometry)	ThermoFisher	Cat# CUST03362	5
Anti-mouse TCRb-143Nd antibody (mass cytometry)	Fluidigm	Cat# 3143010B	1:100 dilution
Anti-FoxP3 antibody (mass cytometry)	ThermoFisher	Cat# 14-5773-82	5
Anti-RORγt antibody (mass cytometry)	BD Biosciences	Cat# 562663	5
Anti-PLZF antibody (mass cytometry)	R&D systems	Cat# MAB29441-100	5
Anti-mouse CD27-150Nd antibody (mass cytometry)	Fluidigm	Cat# 3150017B	1:100 dilution
Anti-mouse CD49d/Integrin a4-151Eu antibody (mass cytometry)	Fluidigm	Cat# 3151016B	1:100 dilution
Anti-mouse TIGIT antibody (mass cytometry)	ThermoFisher	Cat# 16-9501-85	5
Anti-T-bet antibody (mass cytometry)	BioLegend	Cat# 644825	5
Anti-mouse CD152 (CTLA-4) antibody (mass cytometry)	Fluidigm	Cat# 3154008B	1:100 dilution
Anti-IRF4-155Gd antibody (mass cytometry)	Fluidigm	Cat# 3155014B	1:100 dilution
Anti-mouse Ly-108 antibody (mass cytometry)	ThermoFisher	Cat# 14-1508-82	5

Anti-mouse CD279 (PD1)-159Tb antibody (mass cytometry)	Fluidigm	Cat# 3159023B	1:100 dilution
Anti-mouse CD134 (OX-40) antibody (mass cytometry)	BD Biosciences	Cat# 562181	5
Anti-mouse NFIL3 antibody (mass cytometry)	R&D systems	Cat# MAB8888	5
Anti-mouse BCL-6-163Dy antibody (mass cytometry)	Fluidigm	Cat# 3163012B	1:100 dilution
Anti-human/mouse GATA3-167Dy-antibody (mass cytometry)	Fluidigm	Cat# 3167007A	1:100 dilution
Anti-mouse CD278 (ICOS)-168Er antibody (mass cytometry)	Fluidigm	Cat# 3168024B	1:100 dilution
Anti-mouse CD122 antibody (mass cytometry)	ThermoFisher	Cat# 14-1222-82	5
Anti-mouse TGFBR2 antibody (mass cytometry)	ThermoFisher	Cat# PA5-47719	5
Anti-mouse FR4 antibody (mass cytometry)	ThermoFisher	Cat# 16-5446-85	5
Anti-mouse CD223 (LAG-3)-174Yb antibody (mass cytometry)	Fluidigm	Cat# 3174019B	1:100 dilution
Anti-mouse CD127/IL7Ra-175Lu antibody (mass cytometry)	Fluidigm	Cat# 3175006B	1:100 dilution
Anti-APC-176Yb antibody (mass cytometry)	Fluidigm	Cat# 3176007B	1:100 dilution
Anti-mouse CD16/32 antibody (mass cytometry)	BioLegend	Cat# 101335	5
MOG_{38-49}/IA^b tetramer, PE conjugated	Clemente-Casares et al. (2016), Nature 530: 434-440	N/A	5
αGalCer/mCD1d tetramer, APC conjugated	NIH tetramer core facility and this paper	N/A	2.5 or 5
αGalCer/hCD1d tetramer, PE conjugated	MBL	TS-HCG-1	5
**Bacterial and Virus Strains**			
Pathogens: *Listeria monocytogenes*	DMX Corporation, Philadelphia, PA	N/A	
**Human Biological Samples**			
IHL-453	University of Alberta	N/A	
IHL-465	University of Alberta	N/A	
IHL-484	University of Alberta	N/A	
IHL-486	University of Alberta	N/A	
**Chemicals, Peptides, and Recombinant Proteins**			
Streptavidin-PerCP	BD Biosciences	Cat# 554064	
Streptavidin-APC	Agilent Technologies	Cat# PJ252P1-1	
Streptavidin-PE	Agilent Technologies	Cat# PJRS25	

PKH26 Red Fluorescent Cell Linker mini kit	Sigma	Cat# MINI26-1KT	
CellVue^{TM} Claret Far Red mini kit	Sigma	Cat# MINCLARET-1KT	
CFSE (5-(and-6)-Carboxyfluorescein Diacetate, Succinimidyl Ester), mixed isomers	Molecular Probes	Cat# C1157	
NRP-V7 (KYNKANVFL)	Genscript, Amrani et al. (2001), J. Immunol. 167: 655-666	N/A	
MOG_{38-49} (GWYRSPFSRVVH)	Genscript, Clemente-Casares et al. (2016), Nature 530: 434-440	N/A	
KRN 7000	Cayman Chemical Company	Cat# 158021-47-7	
Dinitrophenyl (DNP)-KLH protein Conjugate	Alpha Diagnostic Intl. Inc.	Cat# DNP25-N-10	
Percoll	Sigma-Aldrich	Cat# P1644	
LPS	Sigma-Aldrich	Cat# L2018-5MG	
Heparin solution salt	Sigma-Aldrich	Cat# H3393-250KU	
Red blood cell lysis solution (10X)	Miltenyi Biotec	Cat# 130-094-183	
Maleimide-PEG	Jenkem Tech	Cat# SKU: A3124-10/M-MAL-2000	
$Fe(acac)_3$	Sigma	Cat# F300	
Hematoxylin Gill II	Leica, Surgipath	Cat# 3801521	
Eosin	HARLECO	588X-75	
Picrosirius Red	Sigma-Aldrich	Cat# 365548; CAS: 2610-10-8	
Neutral Buffered Formalin	Sigma	Cat# HT501320-9.5L	
BSA	Sigma	Cat# A4503	
**Commercial Assays**			
CD11c+ microbeads	Miltenyi Biotec	Cat# 130-125-835	
Anti-F4/80 MicroBeads UltraPure, mouse	Miltenyi Biotec	Cat# 130-110-443	
RNeasy Plus Mini Kit	Qiagen	Cat# 74034	
TruSeq Stranded mRNA Sample Prep Kit v2	Illumina	Cat# RS-122-2001	

Reverse transcriptase kit, SuperScript II	Invitrogen	Cat# 18064022	
AMPure XP Beads	Beckman Coulter	Cat# A63882	
KAPA Library Quantification Kit	Kapa Biosystems	Cat# KK4824	
EasySep Mouse B-cell Isolation Kit	Stem Cell Technologies	Cat# 19854	
CD19 enrichment kit	Stem Cell Technologies	Cat# 18954	
BD IMag anti-mCD8 beads	BD Biosciences	Cat# 551516	
BD IMag anti-CD11b beads	BD Biosciences	Cat# 558013	
FITC BrdU Flow Kit	BD Biosciences	Cat# 559619	
PE BrdU Flow Kit	BD Biosciences	Cat# 556029	
Rat Anti-Dinitrophenol (DNP) IgG ELISA Kit, 96 tests, Quantitative	Alpha Diagnostic International	Cat# 650-110-DGG	
InfinityTM ALT (GPT) Liquid Stable Reagent	Thermo Fisher Scientific	Cat# TR71121	
Luminex®	Eve Technologies Corporation	This paper	
IL10 ELISA	R&D Systems	DY417-05	
IL35 ELISA	Biomatik	Cat# EKE61704	
eBioscience™ Foxp3/Transcription Factor Staining Buffer Set	Thermo Fisher Scientific	Cat # 00-5523-00	
eBioscience™ Foxp3/Transcription Factor Fixation/Permeabilization Concentrate and Diluent	Thermo Fisher Scientific	Cat# 00-5521-00	
Fixation/Permeabilization Solution Kit	BD Biosciences	Cat# 554714	
Accu-Chek Aviva Test Strips	Roche	Cat#0645398	
Maxpar X8 Multimetal Labeling Kit	Fluidigm	Cat #201300	
**Deposited Data**			
RNAseq data	This paper	GSE168488	
**Cell Lines**			
FreeStyle™ CHO-S Cells	Thermo Fisher Scientific	Cat# R80007	
**Organisms/Strains**			
Mouse: 8.3-NOD.*G6pc2*$^{-/-}$.*Tcra*$^{-/-}$	Backcrossed and intercrossed at the University of Calgary; Nanjundappa et al. (2017), Cell 171, 655.	N/A	
Mouse: NOD/LtJ	Jackson Laboratory (Bar Harbor, ME)	JAX: 001976	

Mouse: C57BL/6	Jackson Laboratory (Bar Harbor, ME)	JAX: 000664	
Mouse: NOD.*c3c4*	Jackson Laboratory (Bar Harbor, ME)	JAX: 010971	
Mouse: B6.*Cxcr6*^{Gfp}	Jackson Laboratory (Bar Harbor, ME)	JAX: 005693	
Mouse: B6.*IFNγ ARE-Del*$^{-/-}$	Dr. H. Young (NIH, Bethesda, MD) Bae et al. (2016), Hepatology 64, 1189	N/A	
Mouse: B6.*Ifng-ARE-Del*$^{+/-}$.*Cxcr6*$^{GFP/+}$	Intercrossed at the University of Calgary.	N/A	
Mouse: NOD.*c3c4.scid*	Backcrossed and intercrossed at the University of Calgary; Umeshappa et al. (2019), Nat Com 10: 2150	N/A	
Mouse: NODxB6.*IFNγ ARE-Del*$^{-/-}$*)* F1	Intercrossed at the University of Calgary; Umeshappa et al. (2019), Nat Com 10: 2150	N/A	
Mouse: NOD.*Il10*^{tm1Flv} (Tiger)	Intercrossed at the University of Calgary; Clemente-Casares et al. (2016), Nature 530: 434	N/A	
NOD.*B2m*$^{loxP/loxP}$/*B2m*$^{-/-}$	Backcrossed and intercrossed at the University of Calgary; Hamilton-Williams et al. (2003), PNAS 100: 6688.	N/A	

NOD.*B2m*$^{loxP/loxP}$/*B2m*$^{–/–}$.*CD19-Cre*	Backcrossed and intercrossed at the University of Calgary; Nanjundappa et al. (2017), Cell 171, 655; Tsai et al. (2013). J Immunol. 191:70.	N/A	
Ad-hFTCD	Vector Builder; Umeshappa et al. (2019), Nat Com 10: 2150.	N/A	
**Recombinant DNA**			
pMHC-encoding lentiviral DNAs	Vector Builder: Clemente-Casares et al. (2016), Nature 530: 434; this paper	N/A	
