# Supplementary material for: Re-programming mouse liver-resident invariant natural killer T cells for suppressing hepatic and diabetogenic autoimmunity
Source: Nat Commun. 2022 Jun 7;13:3279. doi: 10.1038/s41467-022-30759-w (PMC9174212; doi:10.1038/s41467-022-30759-w)
Supplement: Supplementary file 2 — Description of additional Supplementary File [file 41467_2022_30759_MOESM2_ESM.pdf]

Descriptions of Additional Supplementary data files

Supplementary Data 1: Transcriptional relationships of LiNKT cells with iNKT cell subsets

Supplementary Data 2: Differential gene expression between LiNKT cells from NOD.c3c4 mice and B6 or NOD mice;

Supplementary Data 3: Normalized gene expression counts in LiNKT cells from NOD.c3c4, B6 and NOD mice

Supplementary Data 4: Normalized gene expression counts in LiNKT cells from aGalCer/CD1d-NP-treated vs. control NOD.c3c4 mice

Supplementary Data 5: Normalized gene expression counts in LiNKT cells from aGalCer/CD1d-NP-treated vs. control NOD.c3c4 mice, for the genes listed in Tables 1 and 2

Supplementary Data 6: Transcriptional relationship between aGalCer/CD1d-NP-induced LiNKTR1 cells and pMHCII-NP-induced TR1 CD4+ T
