# Supplementary material for: Re-programming mouse liver-resident invariant natural killer T cells for suppressing hepatic and diabetogenic autoimmunity
Source: Nat Commun. 2022 Jun 7;13:3279. doi: 10.1038/s41467-022-30759-w (PMC9174212; doi:10.1038/s41467-022-30759-w)
Supplement: Supplementary file 9 — Reporting Summary [file 41467_2022_30759_MOESM9_ESM.pdf]

nature portfolio

Corresponding author(s): Pere Santamaria

Last updated by author(s): March 22, 2022

Reporting Summary

Nature Portfolio wishes to improve the reproducibility of the work that we publish. This form provides structure for consistency and transparency in reporting. For further information on Nature Portfolio policies, see our Editorial Policies and the Editorial Policy Checklist.

Statistics

For all statistical analyses, confirm that the following items are present in the figure legend, table legend, main text, or Methods section.

n/a	Confirmed	
☐	☒	The exact sample size (*n*) for each experimental group/condition, given as a discrete number and unit of measurement
☐	☒	A statement on whether measurements were taken from distinct samples or whether the same sample was measured repeatedly
☐	☒	The statistical test(s) used AND whether they are one- or two-sided<br>*Only common tests should be described solely by name; describe more complex techniques in the Methods section.*
☐	☒	A description of all covariates tested
☐	☒	A description of any assumptions or corrections, such as tests of normality and adjustment for multiple comparisons
☐	☒	A full description of the statistical parameters including central tendency (e.g. means) or other basic estimates (e.g. regression coefficient) AND variation (e.g. standard deviation) or associated estimates of uncertainty (e.g. confidence intervals)
☐	☒	For null hypothesis testing, the test statistic (e.g. *F*, *t*, *r*) with confidence intervals, effect sizes, degrees of freedom and *P* value noted<br>*Give P values as exact values whenever suitable.*
☒	☐	For Bayesian analysis, information on the choice of priors and Markov chain Monte Carlo settings
☒	☐	For hierarchical and complex designs, identification of the appropriate level for tests and full reporting of outcomes
☒	☐	Estimates of effect sizes (e.g. Cohen's *d*, Pearson's *r*), indicating how they were calculated

Our web collection on statistics for biologists contains articles on many of the points above.

Software and code

Policy information about availability of computer code

Data collection	Grahpad Prism 6-9, Geneious prime, FlowJo v 10, Cytobank premium, Volocity v7, Partek Flow, Image J v1.44, FastQC, Bowtie 2, Cytoscape 3.2.1, R Studio (UpSetR, pheatmap, Seurat v3).
Data analysis	Grahpad Prism 6-9, FlowJo v10, Cytobank premium, Volocity v7, Partek Flow, Image J v1.44, FastQC, Bowtie 2 (2.2.5), Cytoscape 3.2.1, R Studio (UpSetR, pheatmap, Seurat v3), Cell ranger v3.1, STAR mapper v2.5.3a.

For manuscripts utilizing custom algorithms or software that are central to the research but not yet described in published literature, software must be made available to editors and reviewers. We strongly encourage code deposition in a community repository (e.g. GitHub). See the Nature Portfolio guidelines for submitting code & software for further information.

Data

Policy information about availability of data

All manuscripts must include a data availability statement. This statement should provide the following information, where applicable:

- Accession codes, unique identifiers, or web links for publicly available datasets
- A description of any restrictions on data availability
- For clinical datasets or third party data, please ensure that the statement adheres to our policy

The following datasheets can be found online: Umeshappa_Suppl_Excel_1: Transcriptional relationships of LiNKT cells with iNKT cell subsets; Umeshappa_Suppl_Excel_2: Differential gene expression between LiNKT cells from NOD.c3c4 mice and B6 or NOD mice; Umeshappa_Suppl_Excel_3: Normalized gene expression counts in LiNKT cells from NOD.c3c4, B6 and NOD mice; Umeshappa_Suppl_Excel_4: Normalized gene expression counts in LiNKT cells fromGalCer/C vs. control NOD.c3c4 mice; Umeshappa_Suppl_Excel_5: Normalized gene expression counts in LiNKT cells fromGalCer/CD1d-NP-treated vs. control NOD.c3c4 mice, for the genes listed in Tables 1 and 2; Umeshappa_Suppl_Excel_6: Transcriptional relationship betweenGalCer/CD1d-NP-

induced LiNKTR1 cells and pMHCII-NP-induced TR1 CD4+ T-cells. The raw RNAseq and scRNAseq data files have been uploaded to the GEO database (accession number: GSE168488). Bulk RNA reads were aligned to the GENCODE release 16 of the Mus musculus genome (mm10 assembly). scRNAseq fastq files were processed using either the mouse mm10 or the human GRCh38 human reference transcriptomes.

Field-specific reporting

Please select the one below that is the best fit for your research. If you are not sure, read the appropriate sections before making your selection.

- [x] Life sciences
- [ ] Behavioural & social sciences
- [ ] Ecological, evolutionary & environmental sciences

For a reference copy of the document with all sections, see nature.com/documents/nr-reporting-summary-flat.pdf

Life sciences study design

All studies must disclose on these points even when the disclosure is negative.

Sample size	Based on previous studies in similar biological systems. Sample sizes were as large as possible with tight controls of gender, age, dosing regiments, and experimental conditions within and between experiments, to limit experimental variability. In vitro experiments typically involved smaller sample sizes than in vivo experiments and all statistically significant differences are reported with the corresponding P values.
Data exclusions	No data/mice were excluded from analyses. All analyzed samples are reported.
Replication	Most data sets were replicated in independent experiments and the number of experiments that were pursued are specifically stated in the Figure legends. In addition, the manuscript used several redundant disease model systems as well as read-outs (e.g. similar disease but in a different genetic background) that further substantiate the robustness of the conclusions.
Randomization	Randomization into individual treatment groups was pre-determined. Mice were entered into the study when they reached a pre-determined disease score that ensured sustained disease progression and disease chronicity in all the mice. This information is provided in Methods and Figure legends.
Blinding	Investigators were not blinded to therapeutic outcome as the investigators responsible for treating the mice where generally also those responsible for scoring the outcome of the corresponding experiments. However, they were replicated by different investigators using different read-outs. All cytokine/chemokine determinations were blinded. To minimize potential bias in the scoring of therapeutic effects, pathological scores were evaluated independently by two different investigators (one of whom was not involved in treating the mice or group allocation) and we report the average values, as noted in the manuscript. RNAseq data were generated and allocated to the corresponding groups blindly.

Reporting for specific materials, systems and methods

We require information from authors about some types of materials, experimental systems and methods used in many studies. Here, indicate whether each material, system or method listed is relevant to your study. If you are not sure if a list item applies to your research, read the appropriate section before selecting a response.

Materials & experimental systems

n/a	Involved in the study	
[ ]	[x]	Antibodies
[ ]	[x]	Eukaryotic cell lines
[x]	[ ]	Palaeontology and archaeology
[ ]	[x]	Animals and other organisms
[ ]	[x]	Human research participants
[x]	[ ]	Clinical data
[x]	[ ]	Dual use research of concern

Methods

n/a	Involved in the study	
[x]	[ ]	ChIP-seq
[ ]	[x]	Flow cytometry
[x]	[ ]	MRI-based neuroimaging

Antibodies

Antibodies used

Anti-CD5 antibody, Biotin Conjugated, Clone 53-7.3 BD Biosciences, Cat# 553019;

Anti-CD19 antibody, Phycoerythrin Conjugated, Clone 1D3 BD Biosciences, Cat# 553786

Anti-CD27 antibody, Phycoerythrin Conjugated, Clone LG.3A10 BD Biosciences, Cat# 553235

Anti-CD69 antibody, Biotin Conjugated, Clone H1.2F3, BD Biosciences Cat# 553235

Anti-CD49d antibody, Phycoerythrin Conjugated, Clone 9C10 (MRF4.B) BD Biosciences Cat# 557420

Anti-CD122 antibody, Phycoerythrin Conjugated, Clone TM-beta 1 BD Biosciences Cat# 553362

Anti-CD134 antibody, Biotin Conjugated, Clone OX-86 BD Biosciences Cat# 559862

Anti-CD152 (CTLA-4) antibody, PE Conjugated, Clone UC10-4F10-11 BD Biosciences Cat# 553720

Anti-CD1d antibody, Biotin Conjugated, Clone 1B1 BD Biosciences, Cat# 553844;

Anti-CD11b antibody, FITC Conjugated, Clone M1/70 BD Biosciences, Cat# 557396;

Anti-Mouse CD45R/B220 antibody, BV421 conjugated, Clone RA3-6B2 BD Biosciences, Cat#; 562922

Anti-CD45R/B220 antibody, PerCP Conjugated, Clone RA3-6B2 BD Biosciences, Cat# 553093;

Anti-CD45R/B220 antibody, Allophycocyanin Conjugated, Clone RA3-6B2 BD Biosciences, Cat# 553092

anti-mouse CD223 (LAG-3) antibody, PE conjugated, Clone C9B7W BD Biosciences Cat# 552380

anti-ICOS (CD278) antibody, BV421 conjugated, Clone C398.4A BD Biosciences Cat# 565887

Anti-TCRbeta antibody, FITC Conjugated, Clone H57-597 BD Biosciences Cat# 553171

Anti-PLZF antibody, PE conjugated, Clone R17-809 BD Biosciences Cat# 564850

Anti-human GATA3 Antibody, PE-Cy7 Conjugated, clone L50-823 BD Biosciences Cat# 560405

Anti-Mouse RORγt antibody, PE conjugated, Clone Q31-378 BD Biosciences Cat# 562607

Anti- mouse CD16/CD32 antibody, Unconjugated, Clone 2.4G2 BD Biosciences Cat# 553141

7-AAD Staining Solution, BD Biosciences Cat# 559925

Anti-mouse TIGIT antibody, PE conjugated, Clone GIGD7 Invitrogen Cat# 12-9501-82

Anti-mouse c-MAF antibody, PE conjugated, Clone sym0F1 Invitrogen Cat# 12-9855-42

Anti-mouse CD4 antibody, PE/Cyanine7 conjugated, Clone RM4-5 Biolegend Cat# 100527

Anti-mouse CD8a antibody, PE conjugated, Clone 53-6.7 Biolegend Cat# 100707

Anti-mouse CD279 (PD-1) antibody, PE conjugated, Clone 29F.1A12 Biolegend Cat# 135205

Anti-T-bet antibody, Brilliant Violet 421 conjugated, Clone 4B10 Biolegend Cat# 644815

F4/80 antibody, FITC conjugated, Clone BM8 eBioscience™ Cat# 11-4801-82

F4/80 antibody, APC conjugated, Clone BM8 eBioscience™ Cat# 47-4801-82

Anti-CD19 antibody, PE conjugated, clone 1D3 Pharmingen Cat# 553786

Anti-mouse CD127 antibody, PE conjugated, Clone A7R34 eBioscience™ Cat# 12-1271-82

InVivoMab rat IgG1 isotype control (anti-HRP) antibody, Purified, Clone HRPN Bio X Cell, Cat# BE0088

InVivoMab anti-mouse IL-4 antibody, Clone 11B11 Bio X Cell, Cat# BE0045

InVivoMab anti-mouse IL-10 antibody, Purified, Clone JES5-2A5 Bio X Cell, Cat# BE0049;

InVivoMab anti-mouse IL-21R antibody, Purified, Clone 4A9 Bio X Cell, Cat# BE0258;

InVivoMab anti-mouse/human/rat/monkey/hamster/canine/bovine TGFβ antibody, Purified, Clone 1D11.16.8 Bio X Cell, Cat# BE0057;

InVivoMab anti-mouse IFN- antibody, Clone XMG1.2,Bio X Cell Cat# BE0045

Anti-human TCR-Vα24-Jα18 antibody, APC conjugated, clone 6B11 Biolegend Cat# 342907

Anti-cMAF antibody (mass cytometry) ThermoFisher Cat# CUST03362

Anti-mouse TCRb-143Nd antibody (mass cytometry) Fluidigm Cat# 3143010B

Anti-FoxP3 antibody (mass cytometry) ThermoFisher Cat# 14-5773-82

Anti-RORγt antibody (mass cytometry) BD Biosciences Cat# 562663

Anti-PLZF antibody (mass cytometry) R&D systems Cat# MAB29441-100

Anti-mouse CD27-150Nd antibody (mass cytometry) Fluidigm Cat# 3150017B

Anti-mouse CD49d/Integrin a4-151Eu antibody (mass cytometry) Fluidigm Cat# 3151016B

Anti-mouse TIGIT antibody (mass cytometry) ThermoFisher Cat# 16-9501-85

Anti-T-bet antibody (mass cytometry) BioLegend Cat# 644825

Anti-mouse CD152 (CTLA-4) antibody (mass cytometry) Fluidigm Cat# 3154008B

Anti-IRF4-155Gd antibody (mass cytometry) Fluidigm Cat# 3155014B

Anti-mouse Ly-108 antibody (mass cytometry) ThermoFisher Cat# 14-1508-82

Anti-mouse CD279 (PD1)-159Tb antibody (mass cytometry) Fluidigm Cat# 3159023B

Anti-mouse CD134 (OX-40) antibody (mass cytometry) BD Biosciences Cat# 562181

Anti-mouse NFIL3 antibody (mass cytometry) R&D systems Cat# MAB8888

Anti-mouse BCL-6-163Dy antibody (mass cytometry) Fluidigm Cat# 3163012B

Anti-human/mouse GATA3-167Dy-antibody (mass cytometry) Fluidigm Cat# 3167007A

Anti-mouse CD278 (ICOS)-168Er antibody (mass cytometry) Fluidigm Cat# 3168024B

Anti-mouse CD122 antibody (mass cytometry) ThermoFisher Cat# 14-1222-82

Anti-mouse TGFBR2 antibody (mass cytometry) ThermoFisher Cat# PA5-47719

Anti-mouse FR4 antibody (mass cytometry) ThermoFisher Cat# 16-5446-85

Anti-mouse CD223 (LAG-3)-174Yb antibody (mass cytometry) Fluidigm Cat# 3174019B

Anti-mouse CD127/IL7Ra-175Lu antibody (mass cytometry) Fluidigm Cat# 3175006B

Anti-APC-176Yb antibody (mass cytometry) Fluidigm Cat# 3176007B

Anti-mouse CD16/32 antibody (mass cytometry) BioLegend Cat# 101335

aGalCer/hCD1d tetramer, PE conjugated MBL Cat# TS-HCG-1

Validation

For the purchased antibodies from commercial sources, the authors relied on the species and target cell specificity stated by the Suppliers, under the corresponding catalog number (Suppliers and catalog numbers indicated in the previous section).

Eukaryotic cell lines

Policy information about cell lines

Cell line source(s)	FreeStyle™ CHO-S Cells, Thermo Fisher Scientific Cat# R80007<br>Ad-293 T cells, Clontech Cat# 662269
Authentication	CHO and Ad-293 T cells were commercially available and were not authenticated in our laboratories upon receipt. They were used for pMHC production or adenovirus titration. However, we regularly sequence transduced CHO cell lines (the transduced genes), to confirm identity in terms of transgene specificity.
Mycoplasma contamination	Cell lines used for pMHC production were free of mycoplasma contamination.
Commonly misidentified lines<br>(See ICLAC register)	No commonly misidentified cell lines were used in the study.

Animals and other organisms

Policy information about studies involving animals; ARRIVE guidelines recommended for reporting animal research

Laboratory animals	Mouse strains: NOD/LtJ, C57BL/6 (B6), NOD.c3c4 and B6.Cxcr6Gfp mice were purchased from the Jackson Laboratory (Bar Harbor, ME). 8.3-NOD.G6pc2–/–.Tcra–/– mice have been described 68. B6.Ifng-ARE-Del–/– mice were obtained from H. Young (NIH, Bethesda, MD). NOD.c3c4.scid mice were generated by backcrossing (NOD.c3c4 x NOD.scid) F1 mice with NOD.c3c4 mice for five generations, followed by intercrossing of mice heterozygous for the scid mutation and homozygous for the B6 chromosome 3 and 4 intervals from NOD.c3c4 mice. (NODxB6.Ifng-ARE-Del–/–) F1 mice were generated by intercrossing B6.Ifng-ARE-Del–/– and NOD/LtJ mice. B6.IFNg ARE-Del+/– mice were generated by intercrossing B6.Ifng-ARE-Del–/– and B6 mice. B6.Ifng-ARE-Del+/–.Cxcr6GFP/+ mice were generated by intercrossing B6.Ifng-ARE-Del–/– and B6.Cxcr6Gfp mice. NOD.Il10tm1Flv (Tiger) mice were obtained by backcrossing the Il10tm1Flv allele from C57BL/6.Il10tm1Flv mice (Jackson Lab) onto the NOD/Ltj background for 10 generations. NOD.B2mloxP/loxP/B2m–/– and NOD.B2mloxP/loxP/B2m–/–.CD19-Cre were bred in our laboratory. Age and sex: NOD.c3c4 (~15 weeks-old, males and females); (NODxB6.Ifng-ARE-Del–/–) F1 or Cxcr6eGFP+/– B6.Ifng-ARE-Del+/– mice (~10 week-old females); NOD (6-8 wk-old females for AIH; 13-24 wk-old diabetic females for T1D); 8 wk-old female C57BL/6 for EAE. All mice were housed in specific pathogen-free conditions at room temperature (18-26oC), 40-60% humidity, on 14h light/10h dark cycles.
Wild animals	No wild animals were used in this study.
Field-collected samples	No field collected samples were used in this study.
Ethics oversight	The reported studies were approved by the institutional animal care committee of the Cumming School of Medicine at the University of Calgary.

Note that full information on the approval of the study protocol must also be provided in the manuscript.

Human research participants

Policy information about studies involving human research participants

Population characteristics	The liver isolates studied herein were obtained from 4 patient samples: IHL-453 (61 year-old female with alcoholic liver disease and cirrhosis with past medical history of obesity and type 1 diabetes); IHL-465 (58 year-old female with non-alcoholic steatohepatitis, cirrhosis and an hepatocellular carcinoma, past medical history of hypertension and type 2 diabetes); IHL-484 (38 year-old male with Wilson's disease); and IHL-486 (63 year-old male with resolved hepatitis C virus infection and hepatocellular carcinoma, with past medical history of dyslipidemia).
Recruitment	Patients were recruited at the University of Alberta under informed consent. There was no specific selection for samples of any sex or disease type. Liver explants from which enough T-cell frozen material was available were selected, regardless of other variables.
Ethics oversight	The studies described herein were approved by the institutional ethic boards of both the University of Alberta and the University of Calgary

Note that full information on the approval of the study protocol must also be provided in the manuscript.

Flow Cytometry

Plots

Confirm that:

- [x] The axis labels state the marker and fluorochrome used (e.g. CD4-FITC).
- [x] The axis scales are clearly visible. Include numbers along axes only for bottom left plot of group (a 'group' is an analysis of identical markers).
- [x] All plots are contour plots with outliers or pseudocolor plots.
- [x] A numerical value for number of cells or percentage (with statistics) is provided.

Methodology

Sample preparation	Murine iNKT cells (TCRβint+/aGalCer/CD1d tetramer+) were sorted by flow cytometry from the livers, PCLNs and spleens of aGalCer/CD1d-NP- or Cys-NP-treated NOD.c3c4 mice. B-cells were isolated from PCLN, MLN, spleen, liver and lung mononuclear cell suspensions from aGalCer/CD1d-NP- or Cys-NP-treated mice by cell sorting upon staining with PE-conjugated anti-CD19 mAb or using an Easysep CD19 Positive Selection kit II (Stem Cell Technologies). Briefly, mice were bled to completion by severing the heart and abdominal aortas. Liver cell suspensions were subjected to 37.5% isotonic Percoll gradient (Percoll, Sigma-Aldrich) centrifugation in the presence of heparin (10 U/ml) and mononuclear cells prepared as described above. Lungs were cut into small pieces and digested in RPMI-1640 medium containing 10% FCS, DNAseI (200 U/ml) and Collagenase IV (100 μg/ml) at 37oC for 90 mins. All the pieces were homogenized into a single cell suspension, washed and hemolyzed. Single cell suspensions from liver, lungs and spleen were stained with aGalCer/CD1d tetramer (3 to 5 μg/ml, at room temperature for 1h) and anti-murine TCRβ and B220 mAbs. For iNKTs, TCRβ int+/B220-/tetramer+ cells and for B cells, TCRβ-/B220+ cells were sorted using a FACSAria III instrument (BD Biosciences). Dead cells were excluded from analysis by staining with 7ADD Viability dye from BD biosciences. The percent purity of the iNKT and B-cell preparations were: 88.3+1.9 and 89.9+0.76 for liver iNKTs of control vs. treated mice for bulk RNAseq; 94.6+2.2 and 91.7+0.96 for liver iNKTs of

control vs. treated mice for scRNAseq; 98.1+1 and 100 for liver B-cells of control vs. treated mice for in vitro experiments.

To purify CD11b+ cells, LNs were digested in collagenase D (1.25μg mL-1) and DNAse I (0.1μg mL-1) for 15 min at 37oC, washed, incubated with anti-FcR Abs, and the cell suspensions used to purify CD11b+ cells using anti-CD11b mAb-coated magnetic beads (BD Biosciences). To isolate Kupffer cells (KCs), liver single cell suspensions were subjected to a 37.5% Percoll® gradient centrifugation in the presence of Heparin (10U/ml), to separate RBCs and immune cells, including KCs, from non-immune liver cells. Upon hemolysis of RBCs using a red blood cell lysis solution (Miltenyi Biotec), KCs, were purified using F4/80+ microbeads (Miltenyi Biotec).

Pancreata from NOD mice treated with αGalCer/CD1d-NP or uncoated NP were injected with ~3mL collagenase P (Millipore Sigma Cat# 11213857001, 0.66mg/mL) through the pancreatic duct. The pancreata were then digested at 37ºC for 15 minutes and dispersed with pipetting. The islets were hand-picked under a stereomicroscope and incubated with IL-2-containig LCM for 2 hours in a CO2 incubator. The islet cells and islet infiltrating mononuclear cells were further treated with trypsin at 37ºC for 3 minutes to make single cell suspensions. After Fc blocking, cells were stained with aGalCer/CD1d (for CyTOF) or InsB13-21/IAg7 tetramers at 37ºC or 4oC, respectively, for 60 minutes in the presence of anti-CD4 and anti-B220 antibodies and a viability dye for the last 20 minutes.

For human liver cells, the liver explants were flushed for 20min with PBS to remove red blood cells and other circulating cell types, followed by a 2L hard flush optimized to yield cell mononuclear recoveries from liver that are similar to those obtained after mechanical disruption. The collected cells were then stored in liquid nitrogen. Intra-hepatic liver cell isolates (~20 million/sample) were stained with APC-labeled anti-human TCR-Vα24-Jα18 (1:20 dilution; Clone 6B11, Biolegend) in 100 μL of FACS buffer (1% FBS, 0.1% NaN3 in PBS) for 30min at 4 °C. Stained cells were washed twice in 1 mL FACS buffer, resuspended in 80μL of cold MACS buffer, incubated with 20μL of anti-APC microbeads (Miltenyi Biotec) per 10^7 total cells for 15min at 4°C, and magnetically purified on a MS column according to the manufacturer's protocol. After positive magnetic isolation, the cells were washed and stained with 50μl aGalCer-hCD1d tetramer/antibody staining mix (FITC-labeled mouse anti-human CD3 (Clone HIT3a, Biolegend) at 1:10 dilution plus PE-labeled aGalCer-hCD1d tetramer (MBL) at 1:5 dilution) and incubated at 4 C in the dark for 20min. 7-Aminoactinomycin D (7-AAD, BD Pharmingen) was used to discriminate between live and dead cells. After washing, the cells were resuspended in 500μl FACS buffer and FITC/PE-double-positive cells sorted by flow cytometry.

Instrument

BD LSRII, BD CytoFLEX, BD AriaIII/Fusion (Becton Dickinson, Flow cytometry) and Helios Mass Cytometer (Fluidigm)

Software

FlowJo, Cytobank

Cell population abundance

Cell population abundance is provided as relative values, but the absolute numbers of LiNKT cell subsets can be inferred from the scRNAseq data.

Gating strategy

All samples were gated equally, by identifying the lymphocyte gate in the forward/side scatter plot, excluding cell aggregates, measuring presence of tetramer+ cells using identical gates. The gating strategies used are shown in Supplementary Figures 1, 5 and 6.

☒ Tick this box to confirm that a figure exemplifying the gating strategy is provided in the Supplementary Information.
